# Resting‐state functional connectivity in patients with a complex PTSD or complex dissociative disorder before and after inpatient trauma treatment

**DOI:** 10.1002/brb3.2200

**Published:** 2021-06-09

**Authors:** Yolanda R. Schlumpf, Ellert R. S. Nijenhuis, Carina Klein, Lutz Jäncke, Silke Bachmann

**Affiliations:** ^1^ Division of Neuropsychology, Department of Psychology University of Zurich Zurich Switzerland; ^2^ Clienia Littenheid AG Hospital for Psychiatry and Psychotherapy Littenheid Switzerland; ^3^ Research Unit for Plasticity and Learning of the Healthy Aging Brain University of Zurich Zurich Switzerland; ^4^ Department of Psychiatry, Psychotherapy, and Psychosomatics University Hospitals and University of Halle (Saale) Halle Germany; ^5^ Department of Psychiatry University Hospitals of Geneva Geneva Switzerland

**Keywords:** complex trauma, dissociation, electroencephalography, functional brain network, clinical observation study, resting‐state

## Abstract

**Introduction:**

Recent research suggests that traumatized patients are characterized by disrupted resting‐state functional connectivity. We examined whether neural networks involved in resting‐state change over the course of a phase‐oriented inpatient treatment for complex traumatized and dissociative disorder patients. We also investigated associations between these network alterations and clinical symptoms and emotion regulation skills.

**Methods:**

Pre‐ and post‐treatment, electroencephalography (EEG) was recorded during resting‐state in patients (*n* = 23) with a complex dissociative disorder (CDD) or complex posttraumatic stress disorder (cPTSD). Patients also completed clinical and emotion regulation questionnaires. To reduce variance in the collected data, patients were exclusively tested as one prototypical dissociative part referred to as Apparently Normal Part (ANP). Functional network connectivity was examined and compared with a matched healthy control group (*n* = 37), also measured twice.

**Results:**

Prior to treatment and compared with controls, patients had a significantly lower functional connectivity strength within eyes‐open and eyes‐closed resting‐state networks in the theta and alpha frequency band. Following treatment, functional connectivity strength within these networks was comparable to the control group and comprised areas belonging to the default mode network (DMN) and prefrontal as well as anterior cingulate control regions. Treatment‐related network normalizations in the theta frequency band were associated with a self‐reported increase in the use of cognitive reappraisal strategies and reduction in emotion regulation difficulties.

**Conclusion:**

Phase‐oriented trauma treatment can strengthen resting‐state network connectivity and can increase the capacity of complex traumatized and dissociative patients as ANP to handle emotional challenges effectively.

## Graphical abstract

We examined whether neural networks involved in resting‐state change over the course of an inpatient treatment for complex traumatized and dissociative disorder patients. Prior to treatment and compared with matched‐healthy controls, patients revealed a significantly lower functional network connectivity between regions belonging to the DMN and prefrontal and anterior cingulate areas. Trauma treatment normalized patients' networks, and these changes were associated with an enhanced emotion regulation capacity.

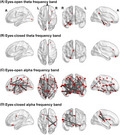

## 1 INTRODUCTION

In‐ and outpatient treatment studies found evidence that long‐term specialized treatment approaches are effective in reducing dissociative and comorbid psychiatric symptoms in complex posttraumatic stress disorder (cPTSD) and dissociative disorder patients (Brand et al., 2009). CPTSD is a diagnosis in ICD‐11 (WHO, 2020). The disorder can arise when an individual has encountered prolonged or repeated traumatic events, such as abuse, violence, or neglect. CPTSD patients have symptoms of PTSD along with the three additional symptom clusters pertaining to emotional dysregulation, interpersonal difficulties, and negative self‐concept (Maercker et al., 2013). Although exposure therapy is beneficial in reducing PTSD symptoms (Schnyder, 2000), clinical observations in complex trauma patients indicate that exposure interventions can have considerable side effects in terms of exacerbation, compliance problems, and high drop‐out rates (Flatten et al., 2004). The current standard of care for cPTSD and dissociative disorders is a phase‐oriented treatment approach (Cloitre et al., 2012; Courtois, 1999; Ford et al., 2005; International Society for the Study of Trauma & Dissociation, 2011; Loewenstein & Welzant, 2010; Steele, Van der Hart, & Nijenhuis, 2001, 2005). Phase‐oriented treatment distinguishes three phases. In the first phase, interventions are geared toward increasing a sense of experienced safety, promoting stabilization, and reducing symptoms. For example, patients learn to manage emotional distress and aversive body sensations. An important goal of the second phase is to integrate traumatic memories. The third phase involves, among others, mourning the traumatic past and enhancing personal growth. The phases involve a recursive spiral more than a linear sequence in that the various treatment phases are typically addressed and readdressed according to the needs and capacities of the patient (Courtois, 1999).

A recent meta‐analysis provided evidence that the phase‐oriented treatment approach is effective in reducing PTSD symptoms in traumatized patients (Corrigan et al., 2020). However, the neural correlates of these therapeutic effects are not yet well understood. To our knowledge, there is only one study to date that addressed functional network changes following a phase‐oriented inpatient treatment in trauma‐related disorders (Schlumpf et al., 2019). After treatment, participants’ neural networks were coupled more. The networks involved lateral prefrontal and anterior cingulate regions, lateral and medial temporal areas (i.e., temporal gyrus, hippocampus, parahippocampal gyrus), and the insula while naturally responding to or cognitively reappraising images that relate to traumatic events. Patients enrolled in this EEG functional connectivity study either fulfilled the diagnostic criteria of a cPTSD or complex dissociative disorder such as dissociative disorder not otherwise specified example 1 (DDNOS‐1) or dissociative identity disorder (DID).

According to the Theory of Structural Dissociation of the Personality (TSDP; Nijenhuis, 2015, 2017; Nijenhuis et al., 2002; Van der Hart et al., 2006), trauma‐related disorders can be described on a continuum from simple PTSD, to cPTSD, to more complex dissociative conditions such as DDNOS‐1 and DID. Within this theory, also PTSD and cPTSD are regarded as dissociative disorders (Nijenhuis, 2014; Van der Hart et al., 2005). Dissociation is defined as a division of the personality in conscious subsystems each with their own first‐person perspective. The severity of a dissociative disorder covaries with the complexity of the division of the personality into dissociative parts. The TSDP suggests there are different prototypical dissociative parts. The basic division is between Apparently Normal Parts (ANP) and Emotional Parts (EPs). A further distinction is made between fragile EPs and controlling EPs (Nijenhuis, 2015, 2017; Van der Hart et al., 2006). To date, fragile EPs and ANPs have been empirically tested in DID patients (Hermans et al., 2006; Reinders et al., 2003, 2006, 2012, 2014, 2016; Schlumpf et al., 2013, 2014; Seidmann et al., 2014). As fragile EP, dissociative patients tend to reenact traumatic memories. In this frame, they may engage in defensive mammalian reactions to perceived threat that can include sympathetic and/or parasympathetic nervous system activation. For example, they may startle, flee, freeze, play dead, or cry for attachment (Nijenhuis, 2015, 2017). ANP is the part that manages daily life activities. As ANP, dissociative patients may have a degree of amnesia for traumatic events, do not or not sufficiently personify traumatic experiences and memories, and attempt to mentally and behaviorally avoid trauma‐related cues and EPs. Neurobiological studies show that within a single DID patient, fragile EP that are typically hyperaroused when confronted with threat cues and that engage in active mammalian defense (e.g., flight) and ANP are associated with distinct brain and autonomic response patterns when exposed to threatening cues (Reinders et al., 2003, 2006, 2014; Schlumpf et al., 2013, 2014). Fragile EP’s reaction pattern is associated with hypo‐activity in prefrontal and anterior cingulate regions that inhibit subcortical limbic areas (i.e., amygdala, insula). Individuals who engage in this pattern typically report re‐experiencing symptoms and are further characterized by autonomic hyperarousal. In contrast, ANP’s reaction pattern is characterized by excessive corticolimbic inhibition and associated autonomic hypoarousal and includes symptoms such as numbness, depersonalization, derealization, and analgesia. These neurobiological findings are consistent with the TSDP’s hypothesis that patients with a trauma‐related disorder react to trauma‐related cues in dissociative part‐dependent psychological and biological ways. To reduce variance in the acquired data, it is, therefore, crucial to control in which part cPTSD or more complex dissociative disorder patients are measured.

A meta‐analysis and systematic review found that PTSD patients show consistently reduced DMN connectivity during resting‐state (Koch et al., 2016). Resting‐state normally activates default mode network (DMN) activity (Fox et al., 2005; Fransson, 2005; Greicius & Menon, 2004; Greicius et al., 2003; Mazoyer et al., 2001). The DMN is a brain network that constitutes of functionally correlated brain areas such as the medial prefrontal cortex (mPFC), posterior cingulate cortex (PCC) in addition to midline parietal structures, lateral parietal regions, and medial and lateral temporal lobes (Buckner et al., 2008; Gusnard & Raichle, 2001; Raichle et al., 2001). A resting‐state perfusion study in DID patients showed abnormal DMN activity compared with a healthy control group (Schlumpf et al., 2014). In PTSD patients, resting‐state connectivity within the DMN correlated negatively with PTSD symptom severity (D. R. Miller et al., 2017; Zhou et al., 2012). A neurofeedback training study with PTSD patients found evidence that this training can enhance DMN connectivity. This increase was associated with enhanced calmness (Kluetsch et al., 2014). In line with these results, a recent fMRI connectivity study in PTSD and DID patients showed that dissociative symptoms can be predicted based on individual DMN functional connectivity scores (Lebois et al., 2020). These findings implicate that a disruption in the DMN is prominent in posttraumatic stress and dissociative disorder patients.

The current study aims to further investigate the neural correlates of treatment‐related changes in patients with chronic and severe interpersonal trauma or dissociative disorder. EEG of patients with cPTSD, DDNOS‐1, or DID was measured in the intracranial space during a resting‐state period that followed exposure to affect‐laden images in the above‐named cognitive reappraisal task (Schlumpf et al., 2019). In contrast to functional magnetic resonance imaging (fMRI), electroencephalography (EEG) offers the possibility to acquire brain functional connectivity at a much higher time resolution. Additionally, it is easier to apply and less aversive than fMRI (Heinrich et al., 2014; Keulers et al., 2015; Mutschler et al., 2014) and, therefore, more convenient for the patients under study. Furthermore, patients completed self‐report questionnaires on clinical symptoms and emotion regulation capacity. As in the cognitive reappraisal study (Schlumpf et al., 2019), participants were instructed to remain in ANP. Experimentally testing patients in various parts would have extended the experiment to a complexity that the patients would not have been able to master. Our primary interest was to explore functional connectivity change for ANP. Apart from this, it is easier to activate an ANP than a fragile EP in an experimental context. We expected pre‐ to post‐treatment functional connectivity increase within the DMN in the patient group.

Resting‐state instructions do not implicate that the patients are really resting. As observed in our previous resting‐state fMRI study, a resting condition can represent a challenge for complex trauma and dissociative disorder patients, particularly when they feel threatened (Schlumpf et al., 2014). This context should not be ignored. We, therefore, assume that the patients’ resting​‐state may have somehow been affected by the preceding emotionally arousing pictures. Based on this assumption, the instruction to relax may lead the patients to divert attention away from the pictures they had seen moments before and from potentially lingering emotional reactions to these cues. Consequently, we may not have measured a neutral affective state but the participants’ ability to relax following the perception of cues that had aroused them emotionally. Based on this line of reasoning, we expect to observe functional connectivity changes in fear‐related neural circuits involving areas implicated in emotion (i.e., insula), cognitive control (i.e., prefrontal cortex [PFC], anterior cingulate cortex [ACC]), and memory (i.e., hippocampus, parahippocampal gyrus). This hypothesis is only subordinate as we do not have a control group that was presented emotionally neutral pictures only. However, it is in line with our previous EEG functional connectivity emotion regulation study (Schlumpf et al., 2019) and fMRI investigations of exposure‐based treatment effects on functional networks in PTSD patients at rest (Shou et al., 2017; Zhu et al., 2018) and during confrontation with reminders of traumatic events (Cisler et al., 2014; Helpman et al., 2016).

Findings on the associations between treatment‐related connectivity changes and changes in self‐reported symptoms have been mixed. Increase in functional connectivity across treatment correlated with PTSD symptom reduction in some studies (Cisler et al., 2014; Zhu et al., 2018). However, other studies could not observe any significant correlation between pre‐ to post‐treatment changes in self‐report instruments and connectivity alterations (Helpman et al., 2016; Schlumpf et al., 2019; Shou et al., 2017). Given these inconsistent findings, we did not formulate a hypothesis on possible relationships between functional connectivity increase and questionnaire data on symptom reduction and/or improvement in emotion regulation.

## 2 METHODS AND MATERIALS

### 2.1 Treatment setting

Patients were recruited from the Psychiatric Hospital Clienia Littenheid AG, Littenheid, Switzerland. They were treated on two specialized wards that provide the same phase‐oriented trauma treatment program. Patients entered psychiatric hospitalization to get intense trauma‐specific care. The majority of patients under investigation was in the first and/or second phase of treatment. The duration of the hospital stay was eight weeks. The treatment concept includes trauma‐adapted and dissociation‐specific psychotherapy (in an individual and group setting), body‐related and cognitive stabilization groups, pharmacotherapy, and other nonverbal treatment modalities (e.g., music, art, occupational therapy). Individual treatment settings are listed in Table S1.

### 2.2 Subjects

In total, 36 patients were enrolled in this project. Nineteen patients fulfilled the criteria of cPTSD that were checked using the *Interview zur Komplexen Posttraumatischen Belastungsstörung* (IK‐PTBS; Sack & Hofmann, 2001). The IK‐PTBS is the German Version of the Structured Interview for Disorders of Extreme Stress (SIDES; Van der Kolk et al., 1992). Seventeen patients fulfilled either the diagnostic criteria of DDNOS‐1 or DID. Clinical diagnoses were checked using the German versions of the Post‐traumatic Diagnostic Scale (PDS; Ehlers et al., 1996) and the Structural Diagnostic Interview for DSM‐IV Dissociative Disorders (SCID‐D; Steinberg, 1993). DDNOS‐1 has become a diagnosis under the name partial DID in ICD‐11 (WHO, 2020). Alternation among dissociative parts aside, the symptoms are not of a different nature in these two disorders, but they are less extreme in DDNOS‐1. Therefore, we merged these two diagnostic categories and refer to this subgroup as complex dissociative disorder (CDD) patients.

All patients had suffered emotional neglect and/or chronic abuse (i.e., sexual, physical, or emotional) starting during childhood. Perpetrators were mainly primary caregivers. Two patients additionally suffered from traumatic war experiences. None of the patients included had recently been further traumatized. All patients were asked to exclusively take part as ANP in the current experiment. After completion of data acquisition, we asked the patients whether they had fulfilled this instruction. They reported that they were able to participate as the intended dissociative part and to prevent unintended switches or coactivations. Comorbid diagnoses and psychotropic medications are listed in Table S2.

Forty mentally healthy participants were enrolled as controls. They were recruited through advertisements posted at the University of Zurich and the psychiatric clinic in Littenheid and through word of mouth. For both groups, we specified regular alcohol and substance abuse, known structural brain damage or neurological disorders as exclusion criteria. For the patient group, we additionally excluded individuals from participation with a comorbid attention deficit disorder, acute suicidality, psychosis, or underweight (Body Mass Index <17). Healthy participants with a lifetime history of any mental disorder were excluded from the study. The PDS (Ehlers et al., 1996) was used to check for potentially traumatic events in the past. Candidate controls who had any traumatic experience and who suffered from post‐traumatic symptoms were not enrolled in the study.

Precocious hospital discharge (three cPTSD patients), inability to undergo the experiment (three CDD patients, four cPTSD patients), low data quality (two healthy controls, two CDD patients), technical issues during data acquisition (one cPTSD patient), and storage failure (one healthy control) caused several drop‐outs. The final sample for data analysis comprised 11 cPTSD, 12 CDD, and 37 healthy controls. Characteristics of patients under investigation are listed in Table 1.

**TABLE 1:** Demographical and clinical data (M, mean; *SD*, standard deviation)

Demographic measures	Patients (*n* = 23)	Controls (*n* = 37)	Group difference (*p*‐value)		
Sex	18 female / 5 male	30 female / 7 male	n.a.		
Education	high school: 70%, college: 30%	high school: 51%, college: 49%	n.a.		
Age (*M* ± *SD*)	42.65 (2.46)	41.46 (2.12)	0.72		

Clinical measures (*M* ± *SD*)	post hoc *t* test				post hoc *t* test				Main effect group		Interaction effect (group × time point)	
	pre	post	*p*‐value	Effect size	pre	post	*p*‐value	Effect size	*p*‐value	Effect size	*p*‐value	Effect size
DERS	112.45 (22.11)	108.22 (24.85)	–	–	55.27 (16.54)	53.54 (14.18)	–	–	<.0001	0.68	.30	0.002
ERQ Reappraisal	20.35 (7.42)	25.21 (6.60)	.0004	−0.7	30.92 (6.43)	31.35 (5.39)	.66	−0.07	<.0001	0.30	.006	0.03
ERQ Suppression	17.26 (5.32)	17.39 (4.05)	–	–	11.56 (4.85)	10.86 (4.42)	––	–	<.0001	0.29	.52	0.002
PCL‐C	58.53 (11.06)	51.62 (11.47)	<.0001	0.61	19.50 (4.06)	19.25 (4.22)	.82	0.06	<.0001	0.84	<.0001	0.04
FDS	26.82 (15.05)	21.71 (14.59)	.0001	0.35	2.34 (2.17)	1.66 (1.80)	.47	0.34	<.0001	0.59	.002	0.02
SDQ‐20	36.79 (10.59)	33.41 (10.22)	.0043	0.33	20.65 (1.34)	20.65 (1.57)	1.00	0.00	<.0001	0.55	.01	0.02
BDI‐II	30.32 (10.13)	25.40 (7.74)	.0002	0.55	2.11 (4.67)	1.64 (3.16)	.62	0.12	<.0001	0.80	.00	0.03
STAI–T	58.11 (8.76)	57.77 (7.61)	–	–	28.73 (6.70)	28.03 (7.77)	–	–	<.0001	0.78	.98	<0.0001

Note

*p*‐values are two‐sided and FDR corrected for post hoc *t* tests. Post hoc *t* tests were only performed if the interaction effect (group × time point) was significant. Effect sizes were calculated as generalized eta^2^ for main and interaction effects and as Cohen's d for *t* tests.

Abbreviations: BDI‐II, Beck's Depression Inventory; DERS, Difficulty in Emotion Regulation Scale; ERQ, Emotion Regulation Questionnaire; FDS, Fragebogen zu Dissoziativen Symptomen; N.a., not applicable; PCL‐C, Post‐traumatic Stress Disorder Checklist, civilian version; post, post‐treatment; pre, pre‐treatment; SDQ‐20, Somatoform Dissociation Questionnaire; STAI‐T, Stait Trait Anxiety Inventory.

The study was approved by the ethics commissions of the cantons Thurgau and Zurich and performed in compliance with the Declaration of Helsinki. Written informed consent was obtained from all participants.

### 2.3 Study design

**FIGURE 1:**
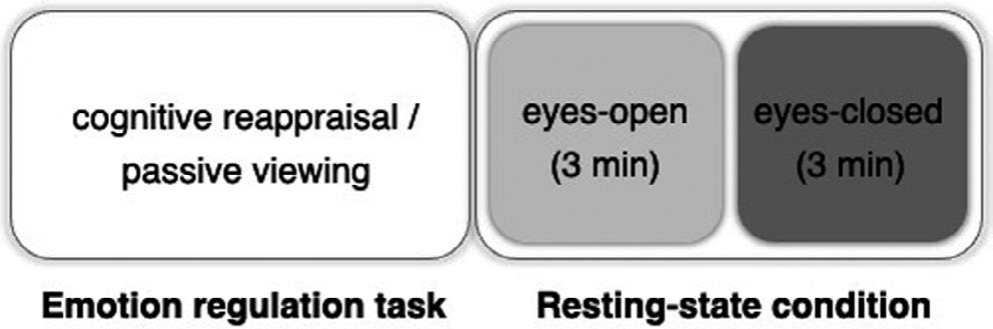
Schematic representation of the experimental setting

We used a longitudinal study design with two time points of data recording. The patient group was assessed at the beginning of the hospital stay (pre‐treatment) and before hospital discharge (post‐treatment; days between pre‐ and post‐treatment: *M* = 41.13, *SD* = 1.44). Controls were also measured twice within a similar time range (days between pre‐ to post‐treatment: *M* = 49, *SD* = 1.28). Each measurement comprised an EEG experiment with several subtasks (see Figure 1) and the assessment of clinical symptoms and emotion regulation capacity using self‐report instruments.

#### 2.3.1 EEG resting‐state experiment

In the present study, we focused on the resting‐state condition that immediately followed the cognitive reappraisal task. During this emotion regulation task, we presented unpleasant and neutral color pictures from the International Affective Picture System (IAPS; Lang et al., 2008), while EEG data were acquired. Investigators’ observations and the participants’ valence and arousal ratings of the stimulus material showed that the pictures, particularly unpleasant images and images comprising of neutral faces or neutral interpersonal interactions, induced negative emotional arousal in the patient group. Details on the cognitive reappraisal task and valence and arousal ratings have been previously published (Schlumpf et al., 2019). Immediately after completion of the emotion regulation task, the EEG signal was registered during both 3 min of eyes‐open and 3 min of eyes‐closed resting‐state (see Figure 1). Subjects were instructed to relax, to stay motionless, and to fixate a yellow dot during the eyes‐open resting‐state to minimize eye movement artifacts.

### 2.4 Questionnaires

#### 2.4.1 Emotion regulation capacity

Pre‐ and post‐treatment, emotion regulation capacity was assessed using two self‐report questionnaires: The German version of the Difficulty in Emotion Regulation Scale (DERS; Gratz & Roemer, 2004) and the German version of the Emotion Regulation Questionnaire (ERQ; Abler & Kessler, 2009). The DERS assesses six different emotion regulation strategies (i.e., non‐acceptance of emotional responses, difficulty in goal‐directed behavior, difficulty controlling impulses, lack of emotional awareness, lack of access to emotion regulation strategies, and lack of emotional clarity). Items are rated on 5‐point scales. The total score consists of all subscales and comprises 36 items (range: 36–180). Higher scores stand for more emotion regulation difficulties. The ERQ is a 10‐item self‐assessment tool and comprises two subscales: cognitive reappraisal and expressive suppression. Items are scored on 7‐point scales (range per subscale: 5–35). The higher the score in a subscale, the more an individual tends to use the corresponding emotion regulation strategy.

#### 2.4.2 Clinical symptoms

Pre‐ and post‐treatment, patients filled in five internationally established self‐report instruments. The civilian version of the Posttraumatic Stress Disorder Checklist (PCL‐C; Teegen, 1997) evaluates the severity of DSM‐IV PTSD symptom criteria using 17 items with a 5‐point scale each. The total score measures intrusion, hyperarousal, and avoidance/numbing (range: 17–85). The *Fragebogen zu Dissoziativen Symptomen* (FDS; Freyberger et al., 1999; Spitzer et al., 1998) comprises 44 items that assess the severity of cognitive‐emotional and several other dissociative symptoms. Participants have to indicate the amount of time (0%–100%) they experience each symptom on the scale. The mean score of the 44 items was calculated (range: 0–100). The Somatoform Dissociation Questionnaire (SDQ‐20; Nijenhuis et al., 1996), a 20‐item self‐report tool, measures the severity of somatoform (i.e., sensorimotor) dissociative symptoms. Items are scored on 5‐point scales (range: 20–100). The severity of depressive symptoms was evaluated using the Beck's Depression Inventory II (BDI‐II; Beck et al., 1996), a 21‐item self‐report questionnaire. Items are rated on 4‐point scales (range: 0–63). Anxiety was obtained with the trait part of the Stait and Trait Anxiety Inventory (STAI‐T; Laux et al., 1981) that consists of 20 items. Items are scored on 4‐point scales (range: 20–80). Details on incomplete data handling in the self‐report questionnaires are reported in the Supplementary Material.

### 2.5 EEG

#### 2.5.1 EEG recording and raw data processing

EEG signals were recorded during 3 min of eyes‐open and eyes‐closed resting‐state using a 64‐channels actiCap system (Brain Products Inc., http://www.brainproducts.com). The average activity of all electrodes served as reference. EEG data were sampled with a BrainVision QuickAmp (Brain Products Inc.) 72‐channel amplifier at 500 Hz with a high‐pass filter of 0.1 Hz, a low‐pass filter of 100 Hz, and a notch filter of 50 Hz. Impedance was controlled before every data acquisition and kept below 25 kOhm. For the raw EEG data preprocessing, we used the BrainVision Analyzer 2.0 software (Brain Products Inc.). An independent component analysis (Jung et al., 2000) was applied to remove eye movement artifacts (i.e., saccades and eye blinks). The data were filtered by employing a band‐pass filter between 0.1 and 40 Hz. We then interpolated bad channels and rejected remaining artifacts (i.e., movement or muscle artifacts) by carrying out the automated raw data inspection in BrainVision Analyzer. This algorithm applied a voltage gradient criterion of 50 μV/ms, an amplitude criterion of ±100 μV, or a low activity criterion of 0.5 μV/100 ms. Finally, data of the 3 min eyes‐open and 3 min eyes‐closed conditions were separately segmented into 2 s epochs. The grand average of these segments was calculated to create topographical distributions of the spectral power values in frequency bands of interest (see Figure S1).

#### 2.5.2 Connectivity analyses in intracranial space

The preprocessed and artifact‐free 2 s segments were transferred to the sLORETA toolbox (Version 20,160,611; https://www.uzh.ch/keyinst/loreta.htm) for functional connectivity analysis in intracranial space (Zalesky et al., 2010). Quality check of the source estimation process is provided in the Supplementary Material of Schlumpf et al. (2019). We calculated intracranial lagged coherence between regions of interest (ROIs) that were defined as the centroid voxels of 84 Brodmann areas (BA, 42 for each hemisphere) implemented in the sLORETA toolbox. Lagged coherence, in contrast to instantaneous measures, reflects pure physiological connectivity as it is resistant to nonphysiological artifacts, particularly to volume conduction and low spatial resolution (Pascual‐Marqui, 2007; Pascual‐Marqui et al., 2011). Description of the corresponding brain region of each ROI is based on visual inspection and on the Juelich Histological and the Harvard‐Oxford cortical/subcortical atlases implemented in the fMRIB software (http://fsl.fmrib.ox.ac.uk/fsl/fslwiki/Atlases). Lagged coherence values were computed separately for the EEG frequency bands delta (1–3.5 Hz), theta (4–8 Hz), alpha (8.5–12 Hz), and beta (12.5–30 Hz; Jäncke et al., 2005; Luck, 2014). The computation calculated matrices of functional connectivity values between all predefined 84 BAs for all single frequency bands and each participant separately.

#### 2.5.3 Network‐based statistical analyses

These 84x84 connectivity matrices from the intracranial analysis in LORETA were further processed in network‐based statistic (NBS, https://www.nitrc.org/projects/nbs/; Zalesky et al., 2010, 2012) using MATLAB (version R2015b, http://www.mathworks.com/). This tool was applied to test for functional connectivity differences in a factorial design. NBS first performs mass‐univariate testing at all connections (i.e., edges) between all ROIs (i.e., nodes). Those connections surviving a predefined sensitivity t‐threshold form supra‐threshold network(s). NBS controls for family‐wise error rate (FWE) by applying nonparametric permutation testing. Based on principles of cluster‐based thresholding of statistical parametric maps (Friston et al., 1994), the size (i.e., all connections) of a supra‐threshold network is tested against the permutation distribution. A *p*‐value indicates the proportion of permutations that yield an equal or larger network compared with the empirical one.

For the purpose of increased power, the CDD and cPTSD patients were merged into one group. We first ran two‐sample *t* tests with the functional connectivity data obtained at the first time point to identify pre‐treatment between‐group network differences. T tests were calculated for each condition (i.e., eyes‐open, eyes‐closed), each frequency band (i.e., delta, theta, alpha, beta), and both contrasts (i.e., patients > controls; controls > patients) separately. A major focus was to track treatment‐related changes in the initially (i.e., pre‐treatment) impaired networks. To this end, we subtracted the lagged coherence post‐treatment values from the lagged coherence pre‐treatment values and calculated two‐sample *t* tests with these difference maps. The difference maps were limited to the networks that showed pre‐treatment group differences. This approach enabled us to investigate group × time point interactions (i.e., treatment‐related network changes). In order to evaluate post‐treatment group differences in the aforementioned networks, we ran additional two‐sample *t* tests. Furthermore, we tested post‐treatment whole‐brain group differences to check for network changes independent from the networks impaired at the first measurement point.

5,000 permutations and a *p*‐value of .05 were applied for all statistical tests in NBS. For the pre‐treatment tests, the highest sensitivity t‐thresholds reaching a significant result of one single network (i.e., network not fallen apart in several components) were chosen. Descending the thresholds in the pre‐treatment analyses in 0.1 steps resulted in significant findings across a large range of thresholds below the highest threshold. Thus, we are confident that the networks presented here represent stable findings. If not otherwise specified, the most liberal sensitivity t‐threshold revealing a significant result was applied for group × time point interactions and post‐treatment group analyses. This approach allowed us to fully exploit whether the initially impaired networks had altered across time. The BrainNet Viewer was used to visualize the functional brain networks (www.nitrc.org/projects/bnv/; Xia et al., 2013).

#### 2.5.4 Association between functional connectivity and emotion regulation capacity

In addition, we correlated treatment‐related changes in networks and emotion regulation capacity. For this purpose, we calculated the mean network strength (i.e., the average of the connectivity values of all edges) of the networks observed in the group × time point NBS analyses. Longitudinal alterations in emotion regulation capacity were evaluated by subtracting the pre‐treatment score of each patient from the corresponding post‐treatment score in the DERS, ERQ Reappraisal, and ERQ Suppression. These difference values (i.e., Diff_DERS, Diff_ERQ_Reappraisal, Diff_ERQ_Suppression) were correlated with the mean functional connectivity values of the networks mentioned above using Kendall's tau correlation. The correlational analyses were restricted to the patient group only. Two‐tailed tests were applied.

#### 2.5.5 Association between functional connectivity and clinical symptoms

The same procedure as described in the last paragraph was conducted with the difference values of the PCL‐C, FDS, SDQ‐20, BDI‐II, and STAI‐T (i.e., Diff_PCL‐C, Diff_FDS, Diff_SDQ‐20, Diff_BDI‐II, Diff_STAI‐T).

#### 2.5.6 Additional statistical analyses and considerations

Besides the NBS analyses, all statistical tests were conducted in R (version 3.4.0, https://www.r‐project.org; R Core Team, 2017). For each self‐report measure, we executed a two (groups) × two (time points) mixed‐design analysis of variance (ANOVA) using the package afex (Singmann et al., 2017). *p*‐values are reported two‐tailed. False discovery rate (FDR) correction was applied in post hoc *t* tests and correlational analyses to adjust for multiple comparisons (Benjamini & Hochberg, 1995).

With the NBS analysis, we conducted several tests on the basis of *p*‐values. The results of these analyses are not interpreted in terms of statistical significance, but as a measure of effect. The *p*‐value is defined as the lowest significance level at which one would still have obtained a significant result for a given data set, significance test, and test problem. This has the advantage that other researchers can decide for themselves whether the results are significant at the significance level they find acceptable (according to Krauth, 1988). Cohen's d, mean, and standard deviation reported for networks revealed by the NBS analysis are based on mean functional connectivity scores of these networks.

## 3 RESULTS

The current EEG study investigates resting‐state functional network changes induced by a phase‐oriented inpatient treatment setting in patients with a diagnosis of cPTSD, DDNOS‐1, or DID.

### 3.1 Network‐based statistics

At the first measurement (pre‐treatment) and compared with healthy controls, patients showed a significantly lower functional connectivity in the theta and alpha frequency band of each condition (eyes‐open theta: *p* = .04, FWE corrected, Cohen's *d* = −0.83, NBS‐specific threshold at *t* = 2.9, patients mean (SD): 0.14(0.09), controls mean (SD): 0.25 (0.16); eyes‐closed theta: *p* = .009, FWE corrected, Cohen's *d* = −1.04, NBS‐specific threshold at *t* = 3.9, patients mean (*SD*): 0.15 (0.11), controls mean (SD): 0.30 (0.16); eyes‐open alpha: *p* = .05, FWE corrected, Cohen's *d *= −0.77, NBS‐specific threshold at *t* = 2.4, patients mean (SD): 0.26 (0.11), controls mean (SD): 0.37 (0.16); eyes‐closed alpha: *p* = .04, FWE corrected, Cohen's *d *= −0.93, NBS‐specific threshold at *t* = 2.8, patients mean (SD): 0.29 (0.10), controls mean (SD): 0.41 (0.14)). The patients’ pre‐treatment hypoconnectivity in the eyes‐open theta network encompassed nine nodes and eight edges in the lateral orbitofrontal cortex (lOFC), precuneus, in right lateralized and medial temporal regions (i.e., inferior temporal gyrus, hippocampus, parahippocampal gyrus), and in the left lateralized occipital pole. During the eyes‐closed condition, the patients’ pre‐treatment hypoconnected network in the theta frequency band involved two right lateralized nodes, one in the lOFC and one in the cingulate gyrus (i.e., dorsal ACC [dACC]/rostral ACC [rACC]), and an edge between the two nodes. In the eyes‐open alpha frequency band network, the patients exhibited a pre‐treatment hypoconnectivity in a network comprising 71 nodes and 122 edges covering the whole cortex. Finally, the patients showed a pre‐treatment hypoconnectivity in the eyes‐closed alpha frequency band network across the prefrontal cortex (i.e., ventrolateral PFC [vlPFC], lOFC), cingulate cortex (i.e., dACC, subgenual ACC [sgACC]), lateral and medial parietal regions (i.e., precuneus, inferior parietal lobule, superior parietal lobule), and the superior temporal gyrus comprising 11 nodes and 11 edges with a right hemispheric predominance.

**FIGURE 2:**
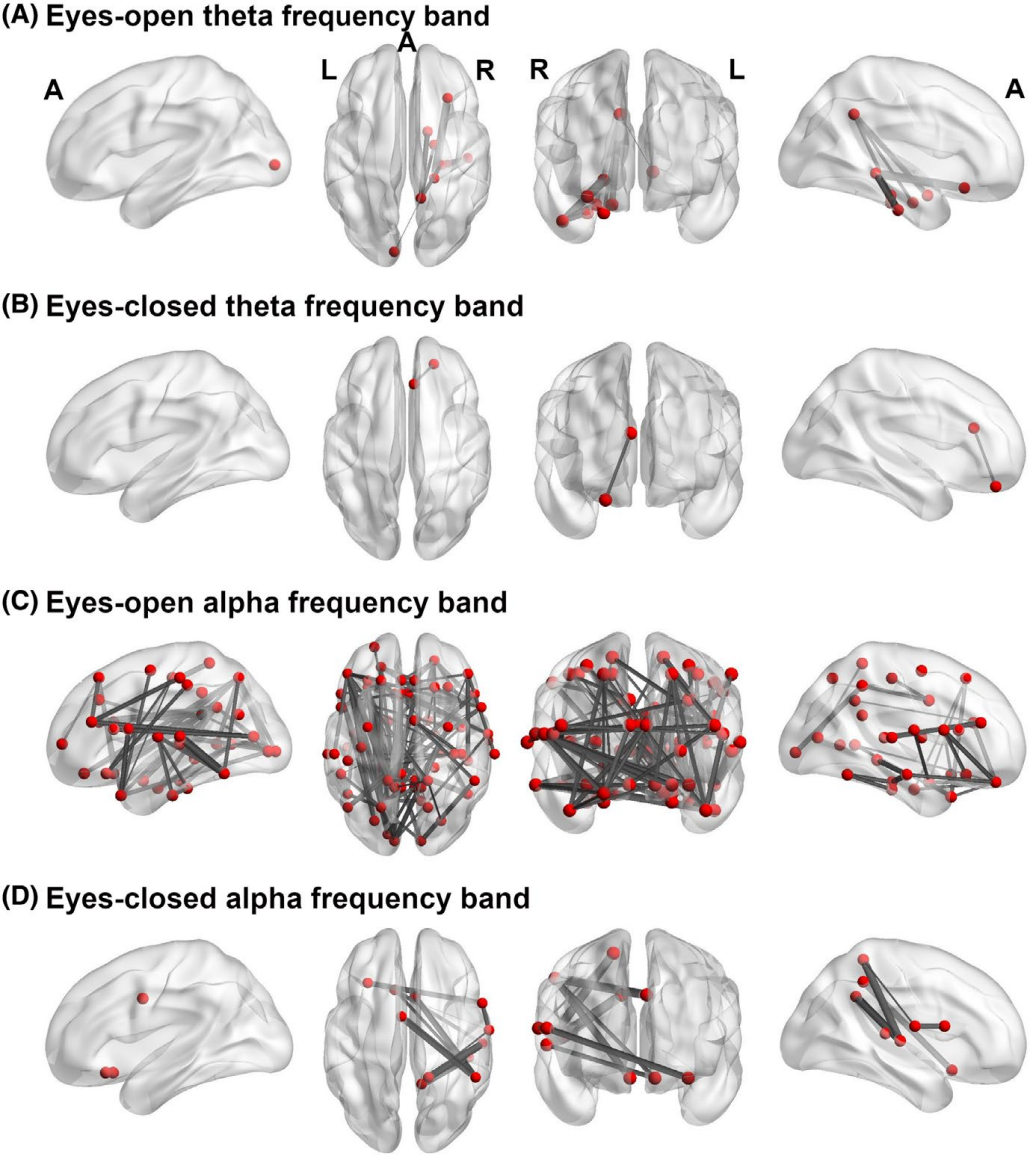
Increase in functional connectivity across treatment within the initially impaired network in the patient group (group × time point interaction) in (a) the theta frequency band during the eyes‐open condition, (b) the theta frequency band during the eyes‐closed condition, (c) the alpha frequency band during the eyes‐open condition, and (d) the alpha frequency band during the eyes‐closed condition. The nodes are depicted as red dots, the gray lines correspond to the connections (i.e., edges). The significance of a connection (i.e., *t*‐value) is indicated by the thickness of the gray line (*p* <.05, FWE corrected). Inter‐ and intrahemispheric connections are shown in left, right, horizontal, and coronal slices. A, anterior, L, left, R, right

To investigate how treatment may have affected functional neural networks, we analyzed group × time point interactions. This analysis revealed a significant interaction effect in the initially impaired networks in the theta and alpha frequency bands in both resting‐state conditions (eyes‐open theta: *p* = .05, FWE corrected, Cohen's *d* = 0.6, NBS‐specific threshold at *t* = 0.8, patients mean (SD): 0.03 (0.09), controls mean (SD): −0.04 (0.12); eyes‐closed theta: *p* = .05, FWE corrected, Cohen's *d* = 0.75, NBS‐specific threshold at *t* = 1.7, patients mean (SD): 0.04 (0.13), controls mean (SD): −0.06 (0.14); eyes‐open alpha: *p* = .03, FWE corrected, Cohen's *d* = 0.4, NBS‐specific threshold at *t* = 0.0, patients mean (SD): 0.03(0.12), controls mean (SD): −0.02 (0.13); eyes‐closed alpha: *p* = .05, FWE corrected, Cohen's *d* = 0.6, NBS‐specific threshold at *t* = 0.4, patients mean (SD): 0.04 (0.13), controls mean (SD): −0.03 (0.13)). Hence, functional connectivity in all initially hypoconnected networks in patients as compared to controls increased across treatment. These networks are depicted in Figure 2. A detailed description of the edges and nodes involved in these networks can be found in Tables S3–S6. Figure S2 depicts the mean functional connectivity change following treatment within the initially impaired networks. Figure S3 outlines individual trajectories in the functional connectivity values from the first to the second measurement point.

Further, we tested for any post‐treatment group differences in the initially hypoconnected networks. At the second time point (post‐treatment), there were no significant group differences anymore (*p* > .05), except in the alpha frequency band of the eyes‐open condition. For comparability reasons, we followed the pre‐treatment t‐threshold of 2.4 and chose the highest post‐treatment sensitivity threshold revealing significant results (*p* = .04, FWE corrected, Cohen's *d* = −0.73, NBS‐specific threshold at *t* = 2.3, patients mean (SD): 0.30 (0.09), controls mean (Sd): 0.37 (0.13)). With six nodes and five edges, the network measured at the second time point is much smaller compared with the network observed at the first time point. Thus, the patients’ connectivity within the eyes‐open alpha frequency band network had become normalized following treatment. However, a subnetwork encompassing nodes in the lOFC, cingulate gyrus (dACC, posterior cingulate gyrus [PCC]), precuneus, and superior temporal gyrus remained hypoconnected (for details see Figure S4 and Table S7).

Finally, we checked for any post‐treatment group differences at the whole‐brain level. There were no significant group differences at the second time point in none of the conditions and none of the frequency bands (*p* > .05).

### 3.2 Association between treatment‐related network and emotion regulation capacity alterations

We investigated the relationship between treatment‐related changes in mean functional connectivity values in the four networks (i.e., eyes‐open theta, eyes‐closed theta, eyes‐open alpha, eyes‐closed alpha) and the changes in self‐reported emotion regulation abilities (i.e., Diff_DERS, Diff_ERQ_Reappraisal, and Diff_ERQ_Suppression) in the patient group. *p*‐values were FDR corrected for each network separately. We found a significant negative correlation between the mean functional connectivity in the eyes‐open theta frequency band network and the Diff_DERS (*p* = .02, FDR corrected, *r*_τ_ = −0.42) and a positive correlation between the mean functional connectivity in the eyes‐closed theta frequency band network and the Diff_ERQ_Reappraisal (*p* = .006, FDR corrected, *r*_τ_ = 0.48). Thus, results in the theta frequency band networks showed that an increase in functional connectivity across treatment is related to an enhanced capacity to regulate emotions and to apply cognitive reappraisal strategies. In the alpha‐band networks, there were no significant correlations between questionnaire and functional connectivity data.

### 3.3 Association between treatment‐related network and clinical symptom alterations

The same correlational approach was applied for self‐reported clinical measures. No kendall's tau correlation survived FDR correction (*p* > .05).

### 3.4 Treatment‐related changes in emotion regulation capacity and clinical symptoms

We ran two (groups) × two (time points) mixed‐design ANOVAs for each self‐report emotion regulation instrument and clinical measure (details are provided in Table 1). As anticipated, the patients showed more difficulties in emotion regulation and more clinical symptoms at each measurement point compared with healthy controls. In addition, we observed a significant increase in the use of reappraisal strategies across treatment in the patient group. Finally, patients exhibited a significant treatment‐related symptom reduction in all clinical domains except trait anxiety.

## 4 DISCUSSION

The present study explored if phase‐oriented treatment is associated with neural network changes in cPTSD, DDNOS‐1, and DID patients. Pre‐ and post‐treatment, EEG‐based functional connectivity was assessed during eyes‐open and eyes‐closed resting‐state. Prior to treatment and compared with a healthy control group, patients had hypoconnected networks in the theta and alpha frequency bands during the eyes‐open and eyes‐closed conditions. In all networks apart from the eyes‐open alpha frequency band network, patients’ hypoconnectivities fully normalized across treatment; thus, group differences largely disappeared post‐treatment. In the eyes‐open alpha frequency band network, patients exhibited a partial recovery over the course of treatment with a subnetwork remaining hypoconnected. Furthermore, pre‐ to post‐treatment network changes in the theta frequency band were related to an improvement in emotion regulation capacity. If not otherwise specified, we refer to these treatment‐related network alterations in the remaining discussion.

In line with our major hypothesis, we found functional network changes in the patients within the DMN that is typically activated at rest (Fox et al., 2005; Fransson, 2005; Greicius & Menon, 2004; Greicius et al., 2003; Mazoyer et al., 2001) and that had previously been identified as being abnormal in traumatized and dissociative patients (Koch et al., 2016; Lebois et al., 2020; Schlumpf et al., 2014). More specifically, we found a connection strengthening in the eyes‐open condition in the theta frequency band including the precuneus, hippocampus, parahippocampal gyrus, and inferior temporal gyrus. The strongest functional connection in this network was observed between the hippocampus and lOFC. Resting‐state functional connectivity increase between regions belonging to the DMN and prefrontal regions at rest has also been observed in PTSD patients following classical (Zhu et al., 2018) or mindfulness‐based exposure therapy (King et al., 2016). Further, we found a significant negative correlation between pre‐ to post‐treatment network changes and a decrease in overall emotion regulation difficulties in the patient group. In the eyes‐closed theta frequency band network, we observed an enhanced connectivity between the lOFC and the dACC/rACC. Remarkably, patients showed a significant relationship between this treatment‐related increase in functional connectivity and an improvement in the use of cognitive reappraisal strategies. Hence, the larger the functional connectivity increase in theta frequency band networks was the larger was the improvement in emotion regulation as measured by different questionnaires.

The eyes‐open alpha frequency band network was large and covered the whole brain. It is, therefore, not feasible to interpret this network node‐ and edge‐wise. To relax and prevent distraction from external cues is more demanding in an eyes‐open compared with an eyes‐closed situation. The large expansion of the eyes‐open network might be related to this additional need for cognitive and concomitant neural resources. Post‐treatment and compared with the healthy control group, a much smaller subnetwork continued to be hypoconnected in the patient group comprising regions that belong to the DMN such as the PCC, precuneus, and superior temporal gyrus (Buckner et al., 2008; Greicius et al., 2003; Raichle et al., 2001) and prefrontal and anterior cingulate control regions including the lOFC and dACC (see Figure S4 and Table S7). The eyes‐closed alpha frequency band network encompassed also different areas of the DMN (i.e., precuneus, superior parietal lobule, inferior parietal lobule, superior temporal gyrus) and prefrontal (i.e., vlPFC) and anterior cingulate (i.e., sgACC) regions.

In summary, patients showed a treatment‐related functional network increase between areas belonging to the DMN and/or PFC and ACC, which is in line with our hypotheses. The DMN has become closely associated with self‐related thinking such as self‐reflection, self‐awareness, or autobiographical memory (Andrews‐Hanna et al., 2010; Buckner et al., 2008; Mason et al., 2007; Northoff et al., 2006). Thus, our results suggest a treatment‐related increase in the capacity to remain in self‐referential states. Interpreting our results in terms of self‐referential processing remains speculation as we did not verify what patients exactly did during resting‐state. However, numerous studies showed maximum theta (Hsieh & Ranganath, 2014; Lomas et al., 2015; Mitchell et al., 2008) and alpha band activity (Benedek et al., 2014; Cooper et al., 2006; Klimesch, 1999; Lomas et al., 2015; Sauseng et al., 2005) during mental tasks that demand sustained internally directed attention such as imagination, meditation, or memory. This observation fits the idea that patients attended to themselves during resting‐state. Neuroimaging data provide evidence that the PFC (E. K. Miller & Cohen, 2001) and ACC (Bush et al., 2000; Stevens et al., 2011; Torta & Cauda, 2011) are activated during tasks that demand emotional and cognitive control. Our subordinate hypothesis was that viewing affect‐laden images prior to a resting‐state condition may influence participants’ ability to relax. The involved images may have elicited affective reactions (e.g., emotional and bodily sensations, movement tendencies, affectively charged thoughts). These reactions may be difficult to inhibit during relaxation, that is, a global task that does not demand or focus attention. The demonstrated involvement of different PFC and ACC subregions is consistent with this hypothesis. The interpretation of an improved capacity to deal with strong affect is further supported by the observation that individuals with a greater increase in theta frequency band network connectivity showed an enhanced use of appropriate emotion regulation strategies following treatment. The partial network recovery in the eyes‐open condition might reflect an intermediate step in treatment where patients as ANP learned to regulate themselves, while they engage in self‐referential thoughts and feelings but did not yet fully reach the level of healthy controls.

The present results provide evidence that a phase‐oriented inpatient trauma treatment approach can change neural circuits that play a key role in trauma‐related disorders. Patients’ pre‐ to post‐treatment network alterations suggest an increased capacity as ANPs to internally focus the attention and to get involved in self‐related thinking. The results might further reflect the patients’ enhanced capacity as ANP to maintain self‐awareness when coping with emotional arousal during resting‐state. Clinical and empirical evidence shows that ANPs typically engage in emotional and mental detachment when they are confronted with cues that are emotional or threatening to them (Hermans et al., 2006; Nijenhuis, 2015, 2017; Reinders et al., 2003, 2006; Schlumpf et al., 2013, 2014; Seidmann et al., 2014; Van der Hart et al., 2006). Most of the patients enrolled in the study were in the first and/or second phase of the phase‐oriented treatment that has a focus on building up grounding techniques and coping skills for difficult feelings and processing of past traumatic memories. One major goal of this phase‐oriented treatment is helping patients as ANP to overcome fear and avoidance of strong effect. This is an essential prerequisite for the integration of trauma memories and development of a cooperative relationship with other dissociative parts (i.e., EPs; Nijenhuis, 2015, 2017; Van der Hart et al., 2006). Thus, our results might reflect a remarkable therapeutic achievement and are in line with our previously published emotion regulation functional connectivity study (Schlumpf et al., 2019) showing that patients as ANP were less depersonalized, derealized, and emotionally numbed following phase‐oriented treatment.

On the behavioral level, patients had achieved a significant treatment‐related reduction in distinct clinical symptoms except for trait anxiety and a significant increase in the use of cognitive reappraisal as an emotion regulation strategy (see Table 1). However, patients as compared to healthy controls still showed more clinical symptoms in all domains after treatment. Network alterations were associated with improvements in emotion regulation capacity, which provides further evidence that therapeutic advancements are associated with neural network changes. However, we could only find a relationship between network alterations and improvement in emotion regulation capacities, but not with symptom reduction. Past studies have also found mixed results (Cisler et al., 2014; Helpman et al., 2016; Schlumpf et al., 2019; Shou et al., 2017; Zhu et al., 2018). Overall and in line with cross‐sectional (Brand, Classen, Lanius, et al., 2009) and longitudinal treatment outcome research (Brand et al., 2013; Jepsen et al., 2014), our results show that a trauma‐ and dissociation‐focused phasic treatment with well‐trained therapists is beneficial for complex traumatized and dissociative patients. As clinical symptoms are still present after treatment and the eyes‐open alpha network has not fully normalized, we would expect further improvements with a continuation of the treatment.

Our study includes several limitations. The current project investigated inpatients treated on two specialized trauma wards. Patient numbers are small and measuring patients in a longitudinal setting requires high investments in time. Therefore, the sample size was relatively small. Further, complex traumatized and dissociative patients show high comorbidity and exclusion of patients using psychotropic medication is not possible. On the other hand, these limitations can also be looked at as a strength because the study conditions were naturalistic. Still, confounding effects of comorbid disorders and psychotropic medication cannot be ruled out. Patients were enrolled when they entered inpatient treatment and we were not able to assign them a waiting or control treatment. Consequently, the present study did not include a waiting list control group. This additional group should be enrolled in future studies in order to assess whether treatment is more effective than simply time alone. Since the patients’ disorders were chronic, it is, however, most unlikely that merely time explains our results. The present study design was limited to two measurement points. A long‐term follow‐up study would be interested to further assess clinical and neural trajectories across a longer time period. Patients were only tested as ANP. Based on the participants self‐reports, we ensured that patients exclusively participated as ANPs. Clinical observations suggest that these self‐reports are quite reliable. However, future studies could think of increasing reliability by taking a video recording of the patients and let the therapists make an additional assessment of the patients’ ability to remain in a specific dissociative part during the experiment. Furthermore, subsequent studies could also test dissociative patients as (different types of) EP. For instance, clinical (Nijenhuis, 2015, 2017; Van der Hart et al., 2006) and empirical observations (Hermans et al., 2006; Reinders et al., 2003, 2006, 2012, 2014, 2016; Schlumpf et al., 2013, 2014; Seidmann et al., 2014) suggest that compared with ANPs, fragile EPs bring forth different affective, cognitive, and behavioral reactions to cues that are of particular importance to them. Thus, testing EPs would allow to further improve our knowledge of neural activation patterns associated with successful therapeutical treatment in complex traumatized and dissociative patients. Last, this clinical observation study investigated the overall effect of a multimodal treatment approach. Hence, the impact of individual treatment modalities cannot be disentangled. Future studies could include fine‐grained measurement scales of treatment success at multiple time points to elucidate which components of the treatment seem to be most important for treatment outcome.

This study is one of the first studies investigating functional connectivity treatment effects associated with a phase‐oriented inpatient trauma treatment approach. After eight weeks of treatment, complex traumatized and dissociative patients achieved improvements in clinical symptoms and emotion regulation capacities and showed a normalization of initially hypoconnected functional brain networks during resting‐state. This increase in functional connectivity was related to an enhanced capacity to react to emotional challenges in adaptive ways.

## CONFLICT OF INTEREST

All authors declare no conflicts of interest.

## AUTHOR CONTRIBUTION

YRS, ERSN, LJ, and SB: contributed to the conception and study design. LJ and SB: supervised the study. YRS performed data acquisition and was responsible for project administration. YRS and CK: analyzed the data and contributed to data visualization. ERSN: assisted with interpretation of findings. YS: wrote the manuscript. All authors critically reviewed content and approved the final version for publication.

## Supporting information

Supplementary Material

## Data Availability

Due to the sensitive nature of the patients enrolled in the study, participants were assured that data would remain confidential and would not be shared.
