# Supplementary material for: Resting‐state functional connectivity in patients with a complex PTSD or complex dissociative disorder before and after inpatient trauma treatment
Source: Brain Behav. 2021 Jun 9;11(7):e02200. doi: 10.1002/brb3.2200 (PMC8323038; doi:10.1002/brb3.2200)
Supplement: Supplementary file 1 — Supplementary Material [file BRB3-11-e02200-s001.pdf]

# SUPPLEMENTARY MATERIAL

# Resting-state functional connectivity in patients with a Complex PTSD or Complex Dissociative Disorder before and after inpatient trauma treatment

Yolanda R. Schlumpf, PhD [1,2§], Ellert R.S. Nijenhuis, PhD [2], Carina Klein, PhD [1], Lutz Jäncke, PhD [1,3], Silke Bachmann, MD [4,5]

**Affiliations:**

[1] Division of Neuropsychology, Institute of Psychology, University of Zurich, Switzerland

[2] Clienia Littenheid AG, Hospital for Psychiatry and Psychotherapy, Littenheid, Switzerland

[3] Research Unit for Plasticity and Learning of the Healthy Aging Brain, University of Zurich, Switzerland

[4] Department of Psychiatry, Psychotherapy, and Psychosomatics, University Hospitals and University of Halle (Saale), Halle, Germany

[5] Department of Psychiatry, University Hospitals of Geneva, Switzerland

[§] Corresponding author

**Correspondence to:**

Yolanda R. Schlumpf, PhD
Division of Neuropsychology
Department of Psychology
University of Zurich
Binzmühlestrasse 14, PO Box 25
8050 Zurich
Switzerland
E-mail: y.schlumpf@psychologie.uzh.ch
Tel: +41 (0) 44 635 74 04
Fax: +41 (0) 44 635 74 09

## METHODS AND MATERIALS

### *Treatment Setting*

Supplementary Table 1 lists individual treatment settings.

**Supplementary Table 1:** Treatment variables and settings per patient included in the analyses (Total n = 23)

| Participant | Diagnosis | INDIVIDUAL | GROUP | STABILISATION | NURSING | PHARMA | BODY | MUSIC | ART | OCCUP | COGNITIVE | DBT | ENJOY |
|---|---|---|---|---|---|---|---|---|---|---|---|---|---|
| 1 | CDD | x | x | x | x | x | x | x | x | | | | |
| 2 | CDD | x | x | x | x | | x | x | x | | | x | |
| 3 | CDD | x | x | x | x | x | x | | x | x | | | x |
| 4 | CDD | x | x | x | x | x | x | x | x | | | | |
| 5 | CDD | x | x | x | x | x | x | | | x | | x | x |
| 6 | CDD | x | x | x | x | | x | | x | x | | x | |
| 7 | CDD | x | x | | x | | x | | x | | | | |
| 8 | CDD | x | x | x | x | x | x | x | x | | | | |
| 9 | CDD | x | x | x | x | | x | | | x | | | |
| 10 | CDD | x | x | x | x | | x | | x | x | | | |
| 11 | CDD | x | x | | x | | x | | x | | | x | |
| 12 | CDD | x | x | x | x | | x | x | x | | | | |
| 13 | cPTSD | x | x | x | x | x | x | x | | x | x | | |
| 14 | cPTSD | x | x | x | x | x | x | x | x | x | | | |
| 15 | cPTSD | x | x | x | x | x | x | x | x | x | | | |
| 16 | cPTSD | x | x | x | x | | | | x | x | | x | |
| 17 | cPTSD | x | x | x | x | | x | x | x | x | | x | |
| 18 | cPTSD | x | x | x | x | | | x | x | | | | |
| 19 | cPTSD | x | x | x | x | x | x | x | x | | | | x |
| 20 | cPTSD | x | x | x | x | | x | x | | x | | x | |
| 21 | cPTSD | x | x | x | x | x | x | x | x | x | | | |
| 22 | cPTSD | x | x | x | x | | x | | x | | | | |
| 23 | cPTSD | x | x | x | x | | x | x | x | | | | |
| **Total** | - | 23 | 23 | 22 | 23 | 10 | 21 | 14 | 19 | 12 | 1 | 7 | 3 |

**CDD**, Complex dissociative disorder; **cPTSD**, Complex posttraumatic stress disorder; **NUMBER**, Number of inpatient stay at the trauma ward (Clienia Littenheid AG); **INDIVIDUAL**, Individual psychotherapy (psychotherapy in a one-to-one setting; eclectic therapeutic approaches were applied); **GROUP**, Group psychotherapy (trauma- and dissociation-specific issues were discussed in a group setting; furthermore, patients received psychoeducation that provided information to better understand and cope with the disorder); **STABILISATION**, Stabilisation groups (involved body-related and/or cognitive therapeutic approaches according to the Psychodynamic Imaginative Trauma Therapy (PITT; Reddemann, 2003); patients were taught grounding and containment skills that enabled them to regulate their emotions); **NURSING**, One-to-one nursing (one nurse took responsibility for all nursing care and was the contact person of a specific patient during the whole inpatient stay); **PHARMA**, Pharmacotherapy (symptoms were treated through the use of drugs); **BODY**, Body-related therapy (physical health was treated in physiotherapy, myofascial therapy, or nordic-walking groups; in addition, movement therapy was applied to increase body perception); **MUSIC**, Music therapy (music was used in a therapeutic setting to address the patients' emotional, cognitive, or physical needs); **ART**, Art therapy (the creative process through

drawing, painting, or designing encouraged patients to express and understand their emotions); **OCCUP**, Occupational therapy (patients were taught self-care, work, and leisure skills to cope with tasks of everyday life); **COGNITIVE**, Cognitive training (particular exercises according to the *Gesellschaft für Gehirntraining e.V.* were performed to improve working memory, memory span, and information processing speed; furthermore, patients learned to evaluate their optimal activation level that allowed them to reach high mental receptiveness); **DBT**, Dialectic behaviour therapy skills group (patients learned about triggers that lead to emotional responses and to apply cognitive and emotional regulation techniques that help to prevent adverse reactions); **ENJOY**, enjoyment group (patients were educated in sensual enjoyment and pleasure).

### *Comorbid Diagnoses and Psychotropic Drugs*

Comorbid diagnoses and psychotropic drugs of the patients were retrieved from their electronic health records (see Supplementary Table 2). Some patients kept benzodiazepine as provisional medication, but none of them took benzodiazepine on a regular basis. In addition, none of the patients under investigation took benzodiazepine at the day of or the day before the EEG experiment.

**Supplementary Table 2:** Comorbid diagnoses and psychotropic medication of the patients included in the analyses (Total n = 23)

| Comorbid diagnoses | Frequency |
|---|---|
| PTSD | 23 |
| Agoraphobia | 1 |
| Panic disorder | 1 |
| Eating disorder | 2 |
| Somatisation disorder | 3 |
| Emotionally unstable personality disorder | 2 |
| Mixed or other personality disorder | 1 |
| Psychopharmaca | Frequency |
| Antidepressiva | 21 |
| Neuroleptics | 5 |
| Antiepileptics | 2 |

PTSD, Posttraumatic Stress Disorder

### *Dealing with Missing Data in Self-Report Instruments*

Considering both groups and each time point, we had the following percentages of incomplete data: 0.37% in the DERS, 0.33% in the ERQ, 0.64% in the PCL-C, 0.44% in the FDS, 0.29% in the SDQ-20, 0.24% in the BDI-II, and 0.71% in the STAI-T. In cases where 90% or more of the responses in a self-report instrument were answered, missing values were replaced by the participant’s mean. For the final data analyses, we had the following missing scores: one

in the DERS (patient group, pre-treatment), one in the ERQ Suppression (control group, pre-treatment), one in the PCL-C (control group, pre-treatment), two in the FDS (patient group, pre-treatment), and one in the STAI-T (patient group, pre-treatment).

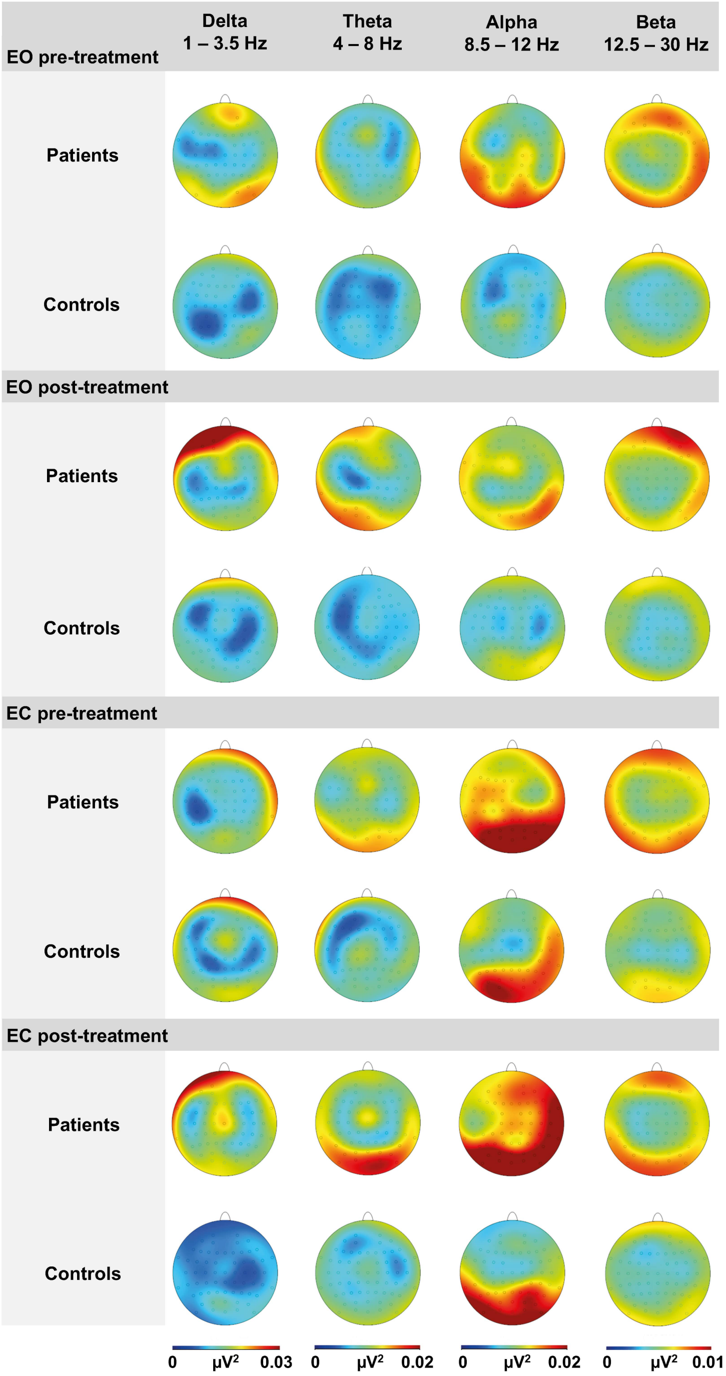


**Supplementary Figure 1**: Topographic mapping of EEG spectral power. The grand average of the preprocessed and segmented EEG data was used for delta, theta, alpha, and beta frequency bands during eyes-closed and eyes-open resting-state at both measurement points for both groups separately. EO, eyes-open; EC, eyes-closed; µ$V^2$= power.

## RESULTS

### *Functional Connectivity*

**Supplementary Table 3:** Patients' functional connectivity increase across treatment in the initially impaired eyes-open theta frequency band network

| Node | | | | Node | | | | | | |
|---|---|---|---|---|---|---|---|---|---|---|
| **BA** | **L/R** | **MNI coordinates (x, y, z)** | **Brain region** | **BA** | **L/R** | **MNI coordinates (x, y, z)** | **Brain region** | **t-value** | **Diff patients** | **Diff controls** |
| 27 | R | (20, -35, -5) | Hippocampus | 47 | R | (30, 25, -15) | Orbitofrontal cortex (lOFC) | 2.56 | 0.02 | -0.07 |
| 20 | R | (45, -20, -30) | Inferior temporal gyrus (Fusiform gyrus) | 27 | R | (20, -35, -5) | Hippocampus | 2.34 | 0.04 | -0.04 |
| 31 | R | (10, -50, 35) | Precuneus | 34 | R | (15, 0, -20) | Parahippocampal gyrus | 2.06 | 0.03 | -0.05 |
| 20 | R | (45, -20, -30) | Inferior temporal gyrus (Fusiform gyrus) | 35a | R | (25, -25, -20) | Parahippocampal gyrus | 2.02 | 0.04 | -0.03 |
| 20 | R | (45, -20, -30) | Inferior temporal gyrus (Fusiform gyrus) | 35b | R | (30, -25, -25) | Parahippocampal gyrus | 1.92 | 0.04 | -0.02 |
| 31 | R | (10, -50, 35) | Precuneus | 35a | R | (25, -25, -20) | Parahippocampal gyrus | 1.79 | 0.02 | -0.02 |
| 28 | R | (20, -10, -25) | Hippocampus | 31 | R | (10, -50, 35) | Precuneus | 1.66 | 0.03 | -0.03 |
| 17a | L | (-10, -90, 0) | Occipital pole (Primary visual cortex) | 31 | R | (10, -50, 35) | Precuneus | 1.24 | -0.01 | -0.05 |

BA, Brodmann area; L, left hemisphere, R, right hemisphere; MNI, Montreal Neurological Institute; Diff patients, difference of mean lagged coherence value in the patients (post-treatment value – pre-treatment value); Diff controls, difference of mean lagged coherence value in controls (post-treatment value – pre-treatment value); lOFC, lateral orbitofrontal cortex. In the sLORETA toolbox, several BAs have two centroid voxels (specified with a and b). NBS-specific threshold at $t = 0.8$, $p < 0.05$ (FWE corrected).

**Supplementary Table 4:** Patients' functional connectivity increase across treatment in the initially impaired eyes-closed theta frequency band network

| Node | | | | Node | | | | | | |
|---|---|---|---|---|---|---|---|---|---|---|
| BA | L/R | MNI coordinates (x, y, z) | Brain region | BA | L/R | MNI coordinates (x, y, z) | Brain region | t-value | Diff patients | Diff controls |
| 11 | R | (20, 45, -20) | Orbitofrontal cortex (lOFC) | 24b | R | (5, 30, 20) | Cingulate gyrus (dACC/rACC) | 2.81 | 0.04 | -0.06 |

BA, Brodmann area; L, left hemisphere, R, right hemisphere; MNI, Montreal Neurological Institute; Diff patients, difference of mean lagged coherence value in the patients (post-treatment value – pre-treatment value); Diff controls, difference of mean lagged coherence value in controls (post-treatment value – pre-treatment value); lOFC, lateral orbitofrontal cortex; dACC, dorsal anterior cingulate cortex; rACC, rostral anterior cingulate cortex. In the sLORETA toolbox, several BAs have two centroid voxels (specified with a and b). NBS-specific threshold at $t = 1.7$, $p < 0.05$ (FWE corrected).

**Supplementary Table 5:** Patients' functional connectivity increase across treatment in the initially impaired eyes-open alpha frequency band network

| Node | | | | Node | | | | | | |
|---|---|---|---|---|---|---|---|---|---|---|
| BA | L/R | MNI coordinates (x, y, z) | Brain region | BA | L/R | MNI coordinates (x, y, z) | Brain region | t-value | Diff patients | Diff controls |
| 37 | L | (-45, -55, -15) | Fusiform gyrus | 42a | L | (-60, -25, 10) | Superior temporal gyrus | 3.33 | 0.08 | -0.04 |
| 41a | L | (-55, -25, 5) | Superior temporal gyrus | 37 | L | (-45, -55, -15) | Fusiform gyrus | 2.73 | 0.08 | -0.02 |
| 20 | L | (-45, -20, -30) | Inferior temporal gyrus (Fusiform gyrus) | 37 | R | (45, -55, -15) | Fusiform gyrus | 2.54 | 0.02 | -0.07 |
| 21 | R | (60, -15, -15) | Middle temporal gyrus | 35a | R | (25, -25, -20) | Parahippocampal gyrus | 2.1 | 0.07 | -0.03 |
| 7 | L | (-20, -65, 50) | Precuneus | 17b | L | (-15, -85, 0) | Lingual gyrus (Primary visual cortex) | 2.06 | 0.03 | -0.04 |
| 17a | L | (-10, -90, 0) | Occipital pole (Primary visual cortex) | 6 | R | (30, -5, 55) | Middle frontal gyrus (Premotor cortex) | 2.05 | 0.04 | -0.04 |
| 5 | L | (-15, -45, 60) | Superior parietal lobule (Paracentral lobule) | 46 | L | (-45, 35, 20) | Middle frontal gyrus (dlPFC) | 2.03 | 0.06 | -0.04 |
| 17a | R | (10, -90, 0) | Occipital pole (Primary visual cortex) | 40 | R | (50, -45, 45) | Inferior parietal lobule (Supramarginal gyrus) | 2 | 0.04 | -0.03 |
| 21 | R | (60, -15, -15) | Middle temporal gyrus | 27 | R | (20, -35, -5) | Hippocampus | 1.96 | 0.05 | -0.02 |
| 28 | L | (-20, -10, -25) | Hippocampus | 30b | L | (-15, -60, 5) | Cingulate gyrus (PCC) | 1.94 | 0.04 | -0.05 |

| | | | | | | | | | | |
|---|---|---|---|---|---|---|---|---|---|---|
| 17b | L | (-15, -85, 0) | Lingual gyrus (Primary visual cortex) | 11 | R | (20, 45, -20) | Orbitofrontal cortex (lOFC) | 1.9 | 0.04 | -0.04 |
| 47 | L | (-30, 25, -15) | Orbitofrontal cortex (lOFC) | 17a | R | (10, -90, 0) | Occipital pole (Primary visual cortex) | 1.83 | 0.03 | -0.03 |
| 31 | L | (-10, -50, 30) | Precuneus | 46 | L | (-45, 35, 20) | Middle frontal gyrus (dlPFC) | 1.81 | 0.07 | -0.01 |
| 41b | R | (45, -30, 10) | Superior temporal gyrus | 46 | R | (45, 35, 20) | Middle frontal gyrus (dlPFC) | 1.81 | 0.04 | -0.03 |
| 39 | L | (-45, -65, 25) | Inferior parietal lobule (Angular gyrus) | 44 | R | (55, 10, 15) | Inferior frontal gyrus (Pars opercularis, vlPFC) | 1.76 | 0.05 | -0.02 |
| 30b | L | (-15, -60, 5) | Cingulate gyrus (PCC) | 35 | L | (-20, -25, -20) | Parahippocampal gyrus | 1.75 | 0.05 | -0.04 |
| 11 | L | (-20, 40, -15) | Orbitofrontal cortex (lOFC) | 42b | R | (60, -10, 15) | Superior temporal gyrus | 1.74 | 0.05 | -0.01 |
| 38 | L | (-40, 15, -30) | Superior temporal gyrus (Temporal pole) | 44 | L | (55, 10, 15) | Inferior frontal gyrus (Pars opercularis, vlPFC) | 1.73 | 0.04 | -0.03 |
| 24b | L | (-5, 30, 20) | Cingulate gyrus (dACC/rACC) | 38 | R | (40, 15, -30) | Superior temporal gyrus (Temporal pole) | 1.69 | 0.03 | -0.05 |
| 11 | R | (20, 45, -20) | Orbitofrontal cortex (lOFC) | 44 | R | (55, 10, 15) | Inferior frontal gyrus (Pars opercularis, vlPFC) | 1.69 | 0.05 | -0.03 |
| 23 | L | (-5, -40, 25) | Cingulate gyrus (PCC) | 33 | L | (-5, 20, 20) | Cingulate gyrus (dACC/rACC) | 1.68 | 0.04 | -0.04 |
| 39 | L | (-45, -65, 25) | Inferior parietal lobule (Angular gyrus) | 45 | R | (50, 20, 15) | Inferior frontal gyrus (Pars triangularis, vlPFC) | 1.68 | 0.05 | -0.01 |
| 30b | L | (-15, -60, 5) | Cingulate gyrus (PCC) | 45 | L | (-50, 20, 15) | Inferior frontal gyrus (Pars triangularis, vlPFC) | 1.67 | 0.01 | -0.06 |
| 30b | L | (-15, -60, 5) | Cingulate gyrus (PCC) | 46 | L | (-45, 35, 20) | Middle frontal gyrus (dlPFC) | 1.67 | 0.05 | -0.02 |
| 11 | L | (-20, 40, -15) | Orbitofrontal cortex (lOFC) | 44 | R | (55, 10, 15) | Inferior frontal gyrus (Pars opercularis, vlPFC) | 1.67 | 0.06 | -0.01 |
| 17a | L | (-10, -90, 0) | Occipital lobe (Primary visual cortex) | 46 | L | (-45, 35, 20) | Middle frontal gyrus (dlPFC) | 1.66 | 0.03 | -0.04 |
| 7 | L | (-20, -65, 50) | Precuneus | 29 | L | (-5, -50, 5) | Cingulate gyrus (PCC) | 1.65 | 0.03 | -0.04 |
| 7 | L | (-20, -65, 50) | Precuneus | 47 | L | (-30, 25, -15) | Orbitofrontal cortex (lOFC) | 1.65 | 0.06 | -0.02 |
| 31 | L | (-10, -50, 30) | Precuneus | 33 | L | (-5, 20, 20) | Cingulate gyrus (dACC/rACC) | 1.64 | 0.05 | -0.03 |
| 31 | L | (-10, -50, 30) | Precuneus | 34 | L | (-15, 0, -20) | Parahippocampal gyrus | 1.63 | 0.04 | -0.03 |
| 7 | L | (-20, -65, 50) | Precuneus | 29 | R | (5, -50, 5) | Cingulate gyrus (PCC) | 1.62 | 0.03 | -0.04 |
| 2a | L | (-55, -25, 50) | Postcentral gyrus (Primary somatosensory cortex) | 46 | L | (-45, 35, 20) | Middle frontal gyrus (dlPFC) | 1.58 | 0.05 | -0.02 |
| 7 | L | (-20, -65, 50) | Precuneus | 11 | L | (-20, 40, -15) | Orbitofrontal cortex (lOFC) | 1.57 | 0.06 | -0.02 |
| 28 | L | (-20, -10, -25) | Hippocampus | 31 | L | (-10, -50, 30) | Precuneus | 1.57 | 0.04 | -0.03 |
| 17b | L | (-15, -85, 0) | Lingual gyrus (Primary visual cortex) | 38 | L | (-40, 15, -30) | Superior temporal gyrus (Temporal pole) | 1.57 | 0.02 | -0.04 |

| | | | | | | | | | | |
|---|---|---|---|---|---|---|---|---|---|---|
| 30b | L | (-15, -60, 5) | Cingulate gyrus (PCC) | 30b | R | (10, -60, 5) | Cuneus | 1.57 | 0.04 | -0.04 |
| 13 | L | (-40, -10, 10) | Insular cortex | 37 | L | (-45, -55, -15) | Fusiform gyrus | 1.56 | 0.02 | -0.06 |
| 8 | L | (-20, 30, 50) | Superior frontal gyrus (dlPFC) | 46 | L | (-45, 35, 20) | Middle frontal gyrus (dlPFC) | 1.55 | 0.05 | -0.02 |
| 11 | R | (20, 45, -20) | Orbitofrontal cortex (lOFC) | 24b | R | (5, 30, 20) | Cingulate gyrus (dACC/rACC) | 1.53 | 0.02 | -0.04 |
| 47 | L | (-30, 25, -15) | Orbitofrontal cortex (lOFC) | 42b | R | (60, -10, 15) | Superior temporal gyrus | 1.52 | 0.06 | 0 |
| 35a | R | (25, -25, -20) | Parahippocampal gyrus | 37 | R | (45, -55, -15) | Fusiform gyrus | 1.51 | 0.02 | -0.04 |
| 8 | R | (20, 25, 50) | Superior frontal gyrus (dlPFC) | 38 | R | (40, 15, -30) | Superior temporal gyrus (Temporal pole) | 1.51 | 0.02 | -0.03 |
| 7 | L | (-20, -65, 50) | Precuneus | 17a | L | (-10, -90, 0) | Occipital pole (Primary visual cortex) | 1.49 | 0.03 | -0.02 |
| 31 | L | (-10, -50, 30) | Precuneus | 25 | R | (5, 15, -15) | Subcallosal cortex (sgACC) | 1.48 | 0.02 | -0.04 |
| 34 | R | (15, 0, -20) | Parahippocampal gyrus | 38 | R | (40, 15, -30) | Superior temporal gyrus (Temporal pole) | 1.48 | 0.03 | -0.04 |
| 13 | L | (-40, -10, 10) | Insular cortex | 20 | L | (-45, -20, -30) | Inferior temporal gyrus (Fusiform gyrus) | 1.46 | 0.03 | -0.04 |
| 28 | L | (-20, -10, -25) | Hippocampus | 25 | R | (5, 15, -15) | Subcallosal cortex (sgACC) | 1.45 | 0.03 | -0.06 |
| 24a | R | (5, 0, 35) | Cingulate gyrus (dACC) | 40 | R | (50, -45, 45) | Inferior parietal lobule (Supramarginal gyrus) | 1.43 | 0.01 | -0.05 |
| 44 | R | (55, 10, 15) | Inferior frontal gyrus (Pars opercularis, vlPFC) | 47 | R | (30, 25, -15) | Orbitofrontal cortex (lOFC) | 1.42 | 0.04 | -0.03 |
| 2b | L | (-45, -30, 45) | Postcentral gyrus (Primary somatosensory cortex) | 47 | L | (-30, 25, -15) | Orbitofrontal cortex (lOFC) | 1.41 | 0.04 | -0.04 |
| 35b | R | (30, -25, -25) | Parahippocampal gyrus | 37 | R | (45, -55, -15) | Fusiform gyrus | 1.39 | 0.02 | -0.04 |
| 42a | R | (65, -25, 10) | Superior temporal gyrus | 46 | R | (45, 35, 20) | Middle frontal gyrus (dlPFC) | 1.38 | 0.02 | -0.03 |
| 46 | L | (-45, 35, 20) | Middle frontal gyrus (dlPFC) | 5 | R | (15, -45, 60) | Superior parietal lobule (Paracentral lobule) | 1.36 | 0.01 | -0.04 |
| 7 | L | (-20, -65, 50) | Precuneus | 37 | L | (-45, -55, -15) | Fusiform gyrus | 1.35 | 0.03 | -0.04 |
| 33 | R | (0, 20, 20) | Cingulate gyrus (dACC/rACC) | 42b | R | (60, -10, 15) | Superior temporal gyrus | 1.35 | 0.04 | -0.01 |
| 8 | L | (-20, 30, 50) | Superior frontal gyrus (dlPFC) | 38 | L | (-40, 15, -30) | Superior temporal gyrus (Temporal pole) | 1.34 | 0.04 | -0.01 |
| 11 | R | (20, 45, -20) | Orbitofrontal cortex (lOFC) | 34 | R | (15, 0, -20) | Parahippocampal gyrus | 1.33 | 0.02 | -0.06 |
| 25 | L | (-10, 20, -15) | Subcallosal cortex (sgACC) | 42b | R | (60, -10, 15) | Superior temporal gyrus | 1.32 | 0.06 | 0 |
| 8 | L | (-20, 30, 50) | Superior frontal gyrus (dlPFC) | 25 | L | (-10, 20, -15) | Subcallosal cortex (sgACC) | 1.29 | 0.05 | -0.02 |
| 40 | L | (-50, -40, 40) | Inferior parietal lobule (Supramarginal gyrus) | 17a | R | (10, -90, 0) | Occipital pole (Primary visual cortex) | 1.29 | 0.03 | -0.02 |

| | | | | | | | | | | |
|---|---|---|---|---|---|---|---|---|---|---|
| 7 | L | (-20, -65, 50) | Precuneus | 25 | R | (5, 15, -15) | Subcallosal cortex (sgACC) | 1.29 | 0.04 | -0.02 |
| 46 | L | (-45, 35, 20) | Middle frontal gyrus (dlPFC) | 44 | R | (55, 10, 15) | Inferior frontal gyrus (Pars opercularis, vlPFC) | 1.29 | 0.04 | -0.01 |
| 9 | L | (-30, 30, 35) | Middle frontal gyrus (dlPFC) | 17a | L | (-10, -90, 0) | Occipital pole (Primary visual cortex) | 1.28 | 0.01 | -0.03 |
| 17b | L | (-15, -85, 0) | Lingual gyrus (Primary visual cortex) | 6 | R | (30, -5, 55) | Middle frontal gyrus (Premotor cortex) | 1.25 | 0.02 | -0.03 |
| 11 | R | (20, 45, -20) | Orbitofrontal cortex (lOFC) | 21 | R | (60, -15, -15) | Middle temporal gyrus | 1.25 | 0.03 | -0.03 |
| 46 | L | (-45, 35, 20) | Middle frontal gyrus (dlPFC) | 25 | R | (5, 15, -15) | Subcallosal cortex (sgACC) | 1.25 | 0.01 | -0.04 |
| 6 | L | (-30, -5, 55) | Middle frontal gyrus (Premotor cortex) | 38 | L | (-40, 15, -30) | Superior temporal gyrus (Temporal pole) | 1.24 | 0.02 | -0.03 |
| 25 | L | (-10, 20, -15) | Subcallosal cortex (sgACC) | 44 | R | (55, 10, 15) | Inferior frontal gyrus (Pars opercularis, vlPFC) | 1.24 | 0.05 | 0 |
| 47 | L | (-30, 25, -15) | Orbitofrontal cortex (lOFC) | 44 | R | (55, 10, 15) | Inferior frontal gyrus (Pars opercularis, vlPFC) | 1.24 | 0.04 | -0.01 |
| 25 | L | (-10, 20, -15) | Subcallosal cortex (sgACC) | 21 | R | (60, -15, -15) | Middle temporal gyrus | 1.23 | 0.04 | -0.02 |
| 34 | R | (15, 0, -20) | Parahippocampal gyrus | 42b | R | (60, -10, 15) | Superior temporal gyrus | 1.23 | 0.04 | 0 |
| 33 | L | (-5, 20, 20) | Cingulate gyrus (dACC/rACC) | 11 | R | (20, 45, -20) | Orbitofrontal cortex (lOFC) | 1.21 | 0.02 | -0.03 |
| 3a | R | (40, -25, 50) | Postcentral gyrus (Primary somatosensory cortex) | 24a | R | (5, 0, 35) | Cingulate gyrus (dACC) | 1.21 | 0.02 | -0.04 |
| 11 | L | (-20, 40, -15) | Orbitofrontal cortex (lOFC) | 31 | L | (-10, -50, 30) | Precuneus | 1.2 | 0.03 | -0.02 |
| 13 | L | (-40, -10, 10) | Insular cortex | 36 | L | (-30, -30, -25) | Parahippocampal gyrus | 1.19 | 0.05 | -0.02 |
| 9 | L | (-30, 30, 35) | Middle frontal gyrus (dlPFC) | 42b | R | (60, -10, 15) | Superior temporal gyrus | 1.16 | 0.06 | 0.02 |
| 7 | L | (-20, -65, 50) | Precuneus | 38 | L | (-40, 15, -30) | Superior temporal gyrus (Temporal pole) | 1.14 | 0.03 | -0.03 |
| 29 | L | (-5, -50, 5) | Cingulate gyrus (PCC) | 38 | R | (40, 15, -30) | Superior temporal gyrus (Temporal pole) | 1.14 | -0.03 | -0.08 |
| 29 | R | (5, -50, 5) | Cingulate gyrus (PCC) | 38 | R | (40, 15, -30) | Superior temporal gyrus (Temporal pole) | 1.14 | -0.03 | -0.08 |
| 25 | L | (-10, 20, -15) | Subcallosal cortex (sgACC) | 31 | L | (-10, -50, 30) | Precuneus | 1.12 | 0.03 | -0.02 |
| 20 | L | (-45, -20, -30) | Inferior temporal gyrus (Fusiform gyrus) | 36 | L | (-30, -30, -25) | Parahippocampal gyrus | 1.1 | 0.05 | -0.03 |
| 25 | R | (5, 15, -15) | Subcallosal cortex (sgACC) | 44 | R | (55, 10, 15) | Inferior frontal gyrus (Pars opercularis, vlPFC) | 1.1 | 0.04 | 0 |
| 20 | L | (-45, -20, -30) | Inferior temporal gyrus (Fusiform gyrus) | 30b | L | (-15, -60, 5) | Cingulate gyrus (PCC) | 1.09 | 0.05 | 0 |
| 31 | L | (-10, -50, 30) | Precuneus | 47 | L | (-30, 25, -15) | Orbitofrontal cortex (lOFC) | 1.09 | 0.05 | 0 |
| 11 | R | (20, 45, -20) | Orbitofrontal cortex (lOFC) | 28 | R | (20, -10, -25) | Hippocampus | 1.09 | 0.02 | -0.04 |

| | | | | | | | | | | |
|---|---|---|---|---|---|---|---|---|---|---|
| 38 | L | (-40, 15, -30) | Superior temporal gyrus (Temporal pole) | 8 | R | (20, 25, 50) | Superior frontal gyrus (dlPFC) | 1.08 | 0.06 | 0.02 |
| 17a | L | (-10, -90, 0) | Occipital pole (Primary visual cortex) | 42b | R | (60, -10, 15) | Superior temporal gyrus | 1.02 | 0.03 | 0 |
| 17b | L | (-15, -85, 0) | Lingual gyrus (Primary visual cortex) | 31 | L | (-10, -50, 30) | Precuneus | 1.01 | 0.01 | -0.03 |
| 11 | R | (20, 45, -20) | Orbitofrontal cortex (lOFC) | 38 | R | (40, 15, -30) | Superior temporal gyrus (Temporal pole) | 0.98 | 0.05 | 0 |
| 45 | L | (-50, 20, 15) | Inferior frontal gyrus (Pars triangularis, vlPFC) | 46 | R | (45, 35, 20) | Middle frontal gyrus (dlPFC) | 0.98 | 0.01 | -0.03 |
| 31 | L | (-10, -50, 30) | Precuneus | 13 | R | (40, -5, 10) | Insular cortex | 0.95 | 0.03 | -0.01 |
| 38 | L | (-40, 15, -30) | Superior temporal gyrus (Temporal pole) | 3a | R | (40, -25, 50) | Postcentral gyrus (Primary somatosensory cortex) | 0.92 | 0.02 | -0.01 |
| 5 | L | (-15, -45, 60) | Superior parietal lobule (Paracentral lobule) | 23 | R | (5, -45, 25) | Cingulate gyrus (PCC) | 0.92 | 0.04 | 0 |
| 38 | L | (-40, 15, -30) | Superior temporal gyrus (Temporal pole) | 6 | R | (30, -5, 55) | Middle frontal gyrus (Premotor cortex) | 0.91 | 0.01 | -0.02 |
| 25 | L | (-10, 20, -15) | Subcallosal cortex (sgACC) | 38 | R | (40, 15, -30) | Superior temporal gyrus (Temporal pole) | 0.91 | 0.01 | -0.04 |
| 24b | L | (-5, 30, 20) | Cingulate gyrus (dACC/rACC) | 11 | R | (20, 45, -20) | Orbitofrontal cortex (lOFC) | 0.89 | 0.01 | -0.03 |
| 17a | L | (-10, -90, 0) | Occipital pole (Primary visual cortex) | 31 | L | (-10, -50, 30) | Precuneus | 0.87 | 0.02 | -0.02 |
| 8 | L | (-20, 30, 50) | Superior frontal gyrus (dlPFC) | 10 | L | (-25, 55, 5) | Frontal pole | 0.86 | 0.04 | -0.01 |
| 31 | L | (-10, -50, 30) | Precuneus | 38 | L | (-40, 15, -30) | Superior temporal gyrus (Temporal pole) | 0.85 | 0.04 | 0 |
| 17a | L | (-10, -90, 0) | Occipital pole (Primary visual cortex) | 23 | L | (-5, -40, 25) | Cingulate gyrus (PCC) | 0.84 | -0.01 | -0.04 |
| 17a | L | (-10, -90, 0) | Occipital pole (Primary visual cortex) | 45 | L | (-50, 20, 15) | Inferior frontal gyrus (Pars triangularis, vlPFC) | 0.84 | 0 | -0.03 |
| 4b | L | (-35, -20, 50) | Precentral gyrus (Primary motor cortex) | 47 | L | (-30, 25, -15) | Orbitofrontal cortex (lOFC) | 0.84 | 0.04 | 0 |
| 31 | L | (-10, -50, 30) | Precuneus | 5 | R | (15, -45, 60) | Superior parietal lobule (Paracentral lobule) | 0.81 | 0.02 | -0.02 |
| 5 | R | (15, -45, 60) | Superior parietal lobule (Paracentral lobule) | 30a | R | (25, -75, 10) | Cuneus | 0.78 | 0 | -0.04 |
| 27 | L | (-20, -35, -5) | Hippocampus | 3b | R | (35, -25, 50) | Postcentral gyrus (Primary somatosensory cortex) | 0.76 | 0.02 | -0.01 |
| 36 | L | (-30, -30, -25) | Parahippocampal gyrus | 3b | R | (35, -25, 50) | Postcentral gyrus (Primary somatosensory cortex) | 0.72 | 0.01 | -0.02 |
| 11 | R | (20, 45, -20) | Orbitofrontal cortex (lOFC) | 29 | R | (5, -50, 5) | Cingulate gyrus (PCC) | 0.71 | -0.01 | -0.05 |
| 8 | R | (20, 25, 50) | Superior frontal gyrus (dlPFC) | 28 | R | (20, -10, -25) | Hippocampus | 0.7 | 0.05 | 0.02 |
| 8 | R | (20, 25, 50) | Superior frontal gyrus (dlPFC) | 34 | R | (15, 0, -20) | Parahippocampal gyrus | 0.7 | 0.05 | 0.02 |
| 23 | L | (-5, -40, 25) | Cingulate gyrus (PCC) | 11 | R | (20, 45, -20) | Orbitofrontal cortex (lOFC) | 0.6 | 0.02 | 0 |
| 31 | R | (10, -50, 35) | Precuneus | 46 | R | (45, 35, 20) | Middle frontal gyrus (dlPFC) | 0.57 | 0.01 | -0.01 |

| | | | | | | | | | | |
|---|---|---|---|---|---|---|---|---|---|---|
| 29 | L | (-5, -50, 5) | Cingulate gyrus (PCC) | 11 | R | (20, 45, -20) | Orbitofrontal cortex (lOFC) | 0.52 | -0.02 | -0.05 |
| 4b | L | (-35, -20, 50) | Precentral gyrus (Primary motor cortex) | 38 | L | (-40, 15, -30) | Superior temporal gyrus (Temporal pole) | 0.48 | -0.01 | -0.03 |
| 28 | L | (-20, -10, -25) | Hippocampus | 3b | R | (35, -25, 50) | Postcentral gyrus (Primary somatosensory cortex) | 0.43 | 0 | -0.02 |
| 35 | L | (-20, -25, -20) | Parahippocampal gyrus | 3b | R | (35, -25, 50) | Postcentral gyrus (Primary somatosensory cortex) | 0.42 | 0.01 | -0.01 |
| 38 | L | (-40, 15, -30) | Superior temporal gyrus (Temporal pole) | 24a | R | (5, 0, 35) | Cingulate gyrus (dACC) | 0.35 | -0.01 | -0.02 |
| 23 | L | (-5, -40, 25) | Cingulate gyrus (PCC) | 47 | L | (-30, 25, -15) | Orbitofrontal cortex (lOFC) | 0.34 | 0.03 | 0.01 |
| 25 | L | (-10, 20, -15) | Subcallosal cortex (sgACC) | 3a | R | (40, -25, 50) | Postcentral gyrus (Primary somatosensory cortex) | 0.3 | 0.02 | 0 |
| 11 | L | (-20, 40, -15) | Orbitofrontal cortex (lOFC) | 7 | R | (15, -65, 50) | Precuneus | 0.29 | 0 | -0.01 |
| 40 | L | (-50, -40, 40) | Inferior parietal lobule (Supramarginal gyrus) | 44 | R | (55, 10, 15) | Inferior frontal gyrus (Pars opercularis, vlPFC) | 0.27 | 0.02 | 0.01 |
| 38 | L | (-40, 15, -30) | Superior temporal gyrus (Temporal pole) | 7 | R | (15, -65, 50) | Precuneus | 0.16 | 0 | -0.01 |
| 47 | L | (-30, 25, -15) | Orbitofrontal cortex (lOFC) | 7 | R | (15, -65, 50) | Precuneus | 0.08 | 0 | 0 |

BA, Brodmann area; L, left hemisphere, R, right hemisphere; MNI, Montreal Neurological Institute; Diff patients, difference of mean lagged coherence value in the patients (post-treatment value – pre-treatment value); Diff controls, difference of mean lagged coherence value in controls (post-treatment value – pre-treatment value); dlPFC, dorsolateral prefrontal cortex; vlPFC, ventrolateral prefrontal cortex; lOFC, lateral orbitofrontal cortex; PCC, posterior cingulate cortex; dACC, dorsal anterior cingulate cortex; rACC, rostral anterior cingulate cortex; sgACC, subgenual anterior cingulate cortex. In the sLORETA toolbox, several BAs have two centroid voxels (specified with a and b). NBS-specific threshold at $t = 0.0$, $p < 0.05$ (FWE corrected).

**Supplementary Table 6:** Patients' functional connectivity increase across treatment in the initially impaired eyes-closed alpha frequency band network

| Node | | | | Node | | | | | | |
|---|---|---|---|---|---|---|---|---|---|---|
| **BA** | **L/R** | **MNI coordinates (x, y, z)** | **Brain region** | **BA** | **L/R** | **MNI coordinates (x, y, z)** | **Brain region** | **t-value** | **Diff patients** | **Diff controls** |
| 31 | R | (10, -50, 35) | Precuneus | 41b | R | (45, -30, 10) | Superior temporal gyrus | 2.22 | 0.04 | -0.03 |
| 41a | R | (55, -20, 5) | Superior temporal gyrus | 31 | R | (10, -50, 35) | Precuneus | 2.11 | 0.04 | -0.04 |
| 5 | R | (15, -45, 60) | Superior parietal lobule (Paracentral lobule) | 41a | R | (55, -20, 5) | Superior temporal gyrus | 2.1 | 0.04 | -0.03 |
| 24a | L | (-5, 0, 35) | Cingulate gyrus (dACC) | 40 | R | (50, -45, 45) | Inferior parietal lobule (Supramarginal gyrus) | 2.1 | 0.04 | -0.05 |
| 25 | R | (5, 15, -15) | Subcallosal cortex (sgACC) | 40 | R | (50, -45, 45) | Inferior parietal lobule (Supramarginal gyrus) | 1.9 | 0.04 | -0.06 |
| 47 | L | (-30, 25, -15) | Orbitofrontal cortex (lOFC) | 44 | R | (55, 10, 15) | Inferior frontal gyrus (Pars opercularis, vlPFC) | 1.8 | 0.04 | -0.03 |
| 42b | R | (60, -10, 15) | Superior temporal gyrus | 44 | R | (55, 10, 15) | Inferior frontal gyrus (Pars opercularis, vlPFC) | 1.61 | 0.11 | -0.01 |
| 25 | L | (-10, 20, -15) | Subcallosal cortex (sgACC) | 41a | R | (55, -20, 5) | Superior temporal gyrus | 1.54 | 0.05 | -0.02 |
| 25 | L | (-10, 20, -15) | Subcallosal cortex (sgACC) | 40 | R | (50, -45, 45) | Inferior parietal lobule (Supramarginal gyrus) | 1.41 | 0.03 | -0.04 |
| 5 | R | (15, -45, 60) | Superior parietal lobule (Paracentral lobule) | 42b | R | (60, -10, 15) | Superior temporal gyrus | 1.18 | 0.02 | -0.03 |
| 47 | L | (-30, 25, -15) | Orbitofrontal cortex (lOFC) | 42b | R | (60, -10, 15) | Superior temporal gyrus | 0.96 | 0.03 | -0.01 |

BA, Brodmann area; L, left hemisphere, R, right hemisphere; MNI, Montreal Neurological Institute; Diff patients, difference of mean lagged coherence value in the patients (post-treatment value – pre-treatment value); Diff controls, difference of mean lagged coherence value in controls (post-treatment value – pre-treatment value); vlPFC, ventrolateral prefrontal cortex; lOFC, lateral orbitofrontal cortex; dACC, dorsal anterior cingulate cortex: sgACC, subgenual anterior cingulate cortex. In the sLORETA toolbox, several BAs have two centroid voxels (specified with a and b). NBS-specific threshold at $t = 0.4$, $p < 0.05$ (FWE corrected).

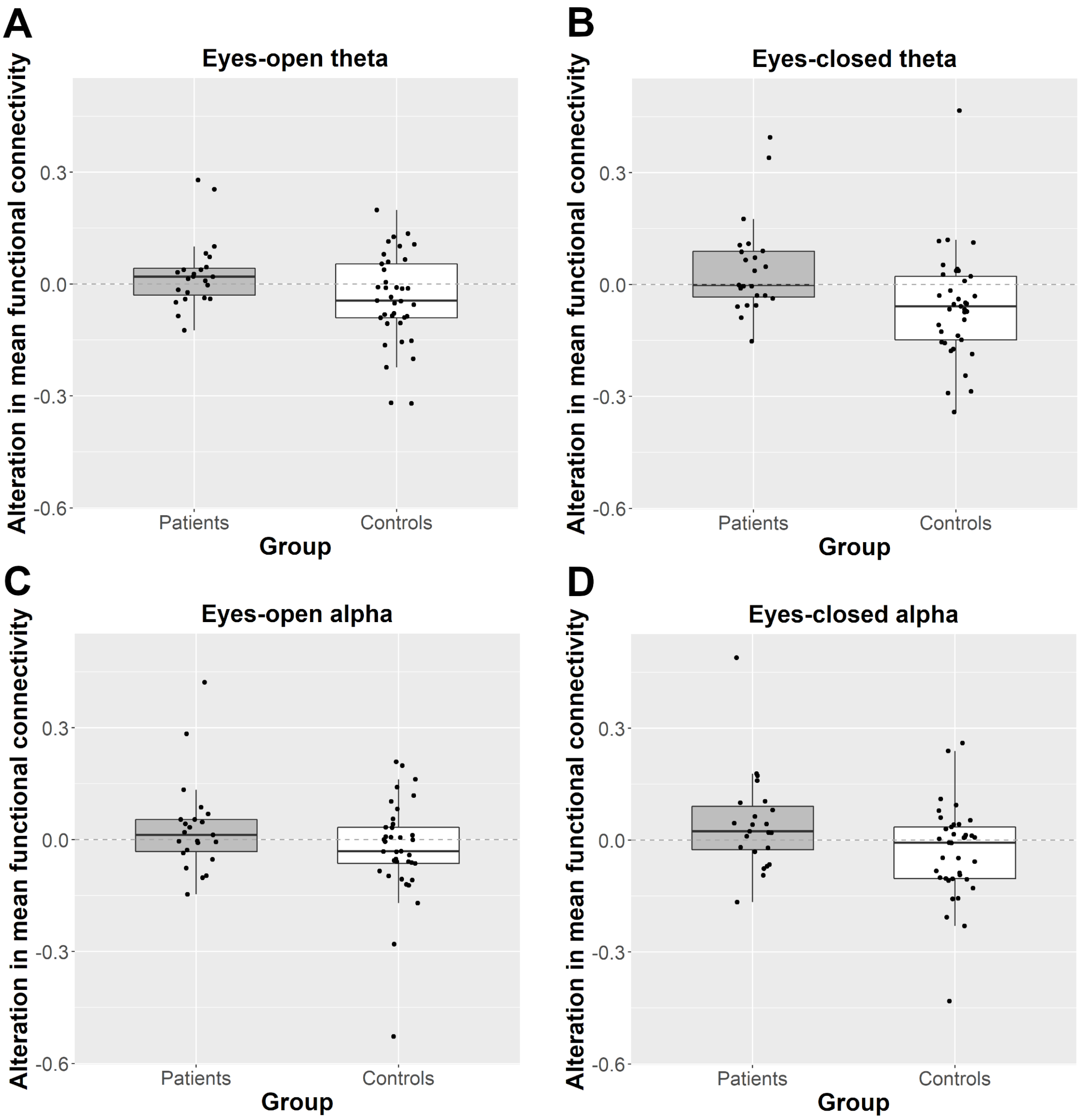


**Supplementary Figure 2**: Functional connectivity change following treatment within the initially impaired network (group x time point interaction) in the A) eyes-open theta frequency band network, B) eyes-closed theta frequency band network, C) eyes-open alpha frequency band network, and D) eyes-closed alpha frequency band network. The graphs outline the mean functional connectivity within the networks revealed by the NBS analysis. A positive value represents higher mean functional connectivity post-treatment compared to pre-treatment, a negative value represents the opposite. Individual values are depicted as black dots. The ends of the whiskers indicate the lowest and highest values within 1.5 x interquartile range (IQR).

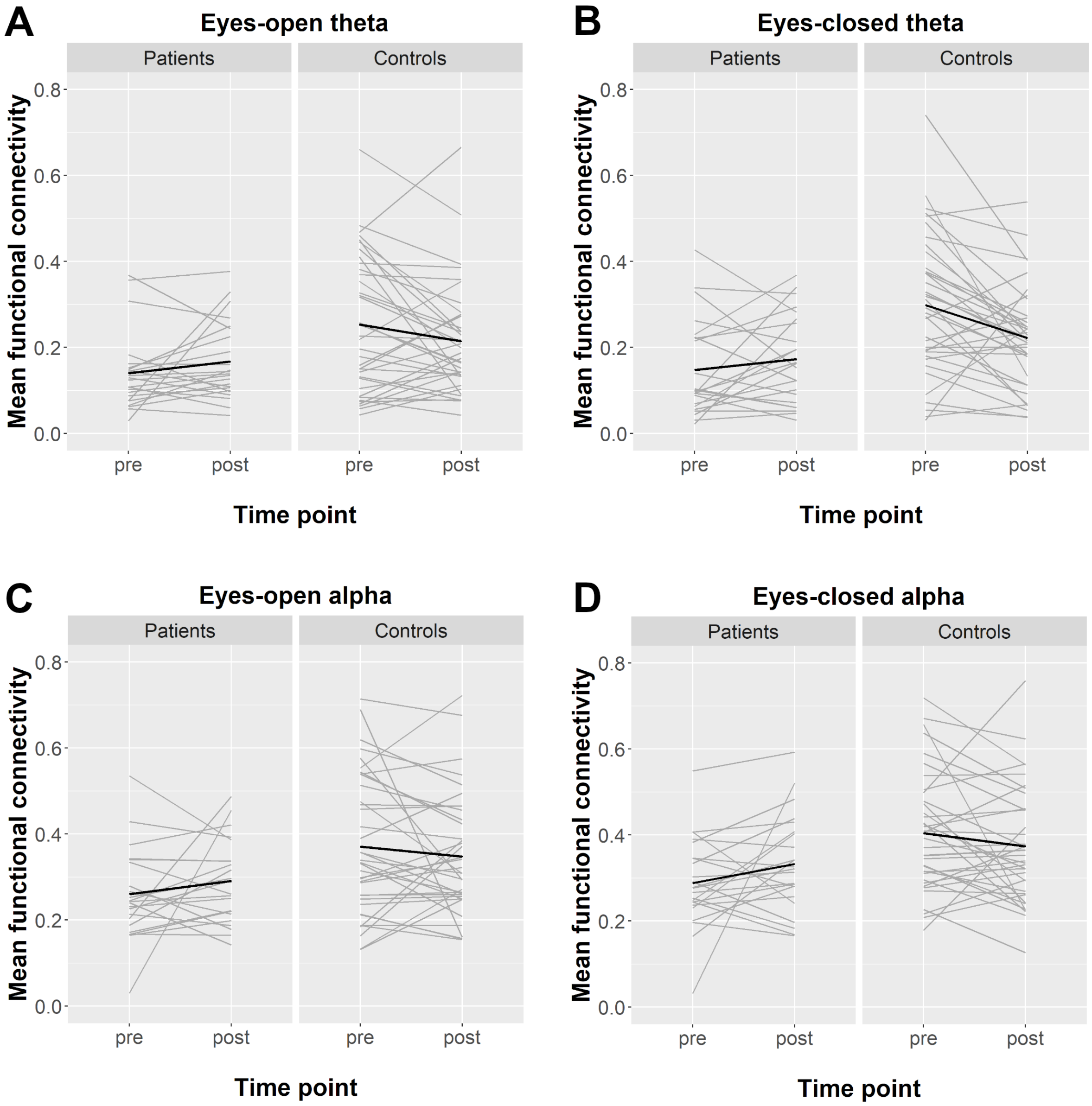


**Supplementary Figure 3:** Pre- to post-treatment trajectories per participant. Mean functional connectivity obtained in the initially impaired networks in the A) eyes-open condition of the theta frequency band, B) eyes-closed condition of the theta frequency band C), eyes-open condition of the alpha frequency band, and D) eyes-closed condition of the alpha frequency band are outlined. For visualisation purposes, the following NBS settings were chosen to extract connectivity values in the non-significant post-treatment comparison: NBS-specific threshold at $t = 0.00$, $p > 0.05$.

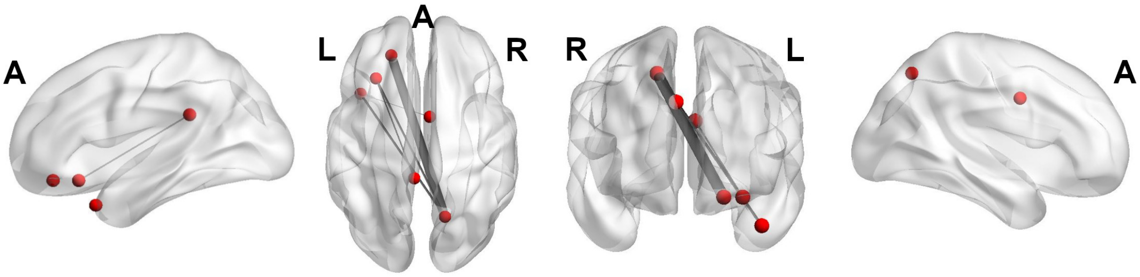


**Supplementary Figure 4:** Patients' post-treatment hypoconnectivity in the initially impaired eyes-open alpha frequency band network. The nodes are depicted as red dots, the gray lines correspond to the connections (i.e., edges). The significance of a connection (i.e., t-value) is indicated by the thickness of the gray line ($p$ < 0.05, FWE corrected). Inter- and intrahemispheric connections are shown in left, right, horizontal, and coronal slices. A, anterior, L, left, R, right.

**Supplementary Table 7:** Patients' post-treatment hypoconnectivity in the initially impaired eyes-open alpha frequency band network

| Node | | | | Node | | | | | | |
|---|---|---|---|---|---|---|---|---|---|---|
| BA | L/R | MNI coordinates (x, y, z) | Brain region | BA | L/R | MNI coordinates (x, y, z) | Brain region | t-value | Diff patients | Diff controls |
| 11 | L | (-20, 40, -15) | Orbitofrontal cortex (lOFC) | 7 | R | (15, -65, 50) | Precuneus | 2.64 | 0.34 | 0.45 |
| 38 | L | (-40, 15, -30) | Superior temporal gyrus (Temporal pole) | 7 | R | (15, -65, 50) | Precuneus | 2.41 | 0.2 | 0.28 |
| 47 | L | (-30, 25, -15) | Orbitofrontal cortex (lOFC) | 7 | R | (15, -65, 50) | Precuneus | 2.39 | 0.22 | 0.3 |
| 23 | L | (-5, -40, 25) | Cingulate gyrus (PCC) | 47 | L | (-30, 25, -15) | Orbitofrontal cortex (lOFC) | 2.38 | 0.42 | 0.52 |
| 38 | L | (-40, 15, -30) | Superior temporal gyrus (Temporal pole) | 24a | R | (5, 0, 35) | Cingulate gyrus (dACC) | 2.35 | 0.21 | 0.27 |

BA, Brodmann area; L, left hemisphere, R, right hemisphere; MNI, Montreal Neurological Institute; Diff patients, difference of mean lagged coherence value in the patients (post-treatment value – pre-treatment value); Diff controls, difference of mean lagged coherence value in controls (post-treatment value – pre-treatment value); lOFC, lateral orbitofrontal cortex; PCC, posterior cingulate cortex; dACC, dorsal anterior cingulate cortex. In the sLORETA toolbox, several BAs have two centroid voxels (specified with a and b). NBS-specific threshold at $t = 2.3$, $p < 0.05$ (FWE corrected).

## REFERENCES

Bradley, M. M., & Lang, P. J. (1994). Measuring emotion: The self-assessment manikin and the semantic differential. *Journal of Behavior Therapy and Experimental Psychiatry*, *25*(1), 49–59. https://doi.org/10.1016/0005-7916(94)90063-9

Reddemann, L. (2003). Die psychodynamisch imaginative Traumatherapie (PITT). *Zeitschrift Für Psychotraumatologie & Psychologische Medizin*, *1*, 1–8.

Schlumpf, Y. R., Nijenhuis, E. R. S., Klein, C., Jäncke, L., & Bachmann, S. (2019). Functional reorganization of neural networks involved in emotion regulation following trauma therapy for complex trauma disorders. *NeuroImage: Clinical*, *23*, 1–14. https://doi.org/10.1016/j.nicl.2019.101807
